# Pluripotent stem cells related to embryonic disc exhibit common self-renewal requirements in diverse livestock species

**DOI:** 10.1242/dev.199901

**Published:** 2021-12-07

**Authors:** Masaki Kinoshita, Toshihiro Kobayashi, Benjamin Planells, Doris Klisch, Daniel Spindlow, Hideki Masaki, Susanne Bornelöv, Giuliano Giuseppe Stirparo, Hitomi Matsunari, Ayuko Uchikura, Ismael Lamas-Toranzo, Jennifer Nichols, Hiromitsu Nakauchi, Hiroshi Nagashima, Ramiro Alberio, Austin Smith

**Affiliations:** 1 Wellcome-MRC Cambridge Stem Cell Institute, Jeffery Cheah Biomedical Centre, University of Cambridge, Cambridge CB2 0AW, UK; 2 Center for Genetic Analysis of Behavior, National Institute for Physiological Sciences, Okazaki, Aichi 444-8787, Japan; 3 Division of Mammalian Embryology, Centre for Stem Cell Biology and Regenerative Medicine, Institute of Medical Science, The University of Tokyo, Minato-ku, Tokyo 108-8639, Japan; 4 School of Biosciences, University of Nottingham, Sutton Bonington Campus, Nottingham LE12 5RD, UK; 5 Living Systems Institute, University of Exeter, Stocker Road, Exeter EX4 4QD, UK; 6 Division of Stem Cell Therapy, Distinguished Professor Unit, Institute of Medical Science, The University of Tokyo, Minato-ku, Tokyo 108-8639, Japan; 7 Laboratory of Medical Bioengineering, Department of Life Sciences, School of Agriculture, Meiji University, 1-1-1 Higashi-mita, Tama, Kawasaki 214-8571, Japan; 8 Department of Physiology, Development and Neuroscience, University of Cambridge, Cambridge CB2 1GA, UK; 9 Institute for Stem Cell Biology and Regenerative Medicine, Department of Genetics, Stanford University School of Medicine, Stanford, CA 94305 USA

**Keywords:** Embryonic stem cells, Epiblast, Mammalian embryo, Nuclear transfer, Pluripotency, Self-renewal

## Abstract

Despite four decades of effort, robust propagation of pluripotent stem cells from livestock animals remains challenging. The requirements for self-renewal are unclear and the relationship of cultured stem cells to pluripotent cells resident in the embryo uncertain. Here, we avoided using feeder cells or serum factors to provide a defined culture microenvironment. We show that the combination of activin A, fibroblast growth factor and the Wnt inhibitor XAV939 (AFX) supports establishment and continuous expansion of pluripotent stem cell lines from porcine, ovine and bovine embryos. Germ layer differentiation was evident in teratomas and readily induced *in vitro*. Global transcriptome analyses highlighted commonality in transcription factor expression across the three species, while global comparison with porcine embryo stages showed proximity to bilaminar disc epiblast. Clonal genetic manipulation and gene targeting were exemplified in porcine stem cells. We further demonstrated that genetically modified AFX stem cells gave rise to cloned porcine foetuses by nuclear transfer. In summary, for major livestock mammals, pluripotent stem cells related to the formative embryonic disc are reliably established using a common and defined signalling environment.

This article has an associated ‘The people behind the papers’ interview.

## Abstract (precis)

**Summary:** We report the derivation of similar, stable and continuously expandable pluripotent stem cells related to embryonic disc epiblast from embryos of pig, sheep and cow.

## INTRODUCTION

Pluripotent stem cell (PSC) lines have been established from rodent and primate embryos (Brons et al., 2007; Evans and Kaufman, 1981; Martin, 1981; Tesar et al., 2007; Thomson et al., 1998, 1995) and extensively characterised. These cells correspond to transient phases of early embryo development, yet exhibit sustained self-renewal in culture. Mouse and rat embryonic stem (ES) cells can be reintroduced to pre-implantation embryos and contribute extensively to chimaeric animals, including to the germline (Bradley et al., 1984; Buehr et al., 2008; Li et al., 2008). Consequently, mouse ES cells have proven a revolutionary tool for gene modification and complex genome engineering in mammals. Human embryo-derived and induced pluripotent stem cells (PSCs), on the other hand, are widely used to model early human embryo development (Rossant and Tam, 2021) and to generate differentiated cell types and tissues for disease modelling and cell therapy (Yamanaka, 2020). Availability of comparably stable and operable PSCs from livestock mammals would potentiate production of genetically enhanced farm animals. PSCs from these species would also constitute a valuable resource for basic and biomedical research in areas including comparative developmental biology (Kobayashi et al., 2017), xenotransplantation and genetic modification of animal hosts for production of transplantable human tissues and organs (Masaki and Nakauchi, 2017). In addition, differentiation-competent livestock PSCs would provide a renewable platform for sustainable manufacturing of cell-derived meat and other products (Post et al., 2020), an area of emerging interest.

Progress in establishing stable cultures of PSCs from farm animals, whether from embryos or by molecular reprogramming, has lagged behind mouse and human (Ezashi et al., 2016). Attempts to derive pluripotent stem cells from large animal embryos initially focused on recapitulating the derivation of mouse ES cells (Piedrahita et al., 1990; Saito et al., 1992; Wianny et al., 1997). However, the capture of naïve stem cells appears to depend on species-specific culture conditions (Boroviak et al., 2015; Buehr et al., 2008; Dong et al., 2019; Guo et al., 2016; Li et al., 2008) and insight remains limited into the requirements for animals other than rodents and human. To date, there are no convincing reports of naïve pluripotent stem cells analogous to mouse ES cells derived from livestock. In contrast, recent studies have described embryo-derived stem cell lines from pig (Choi et al., 2019; Gao et al., 2019), cow (Bogliotti et al., 2018; Zhao et al., 2021) and sheep (Vilarino et al., 2020) that exhibit features referred to as either primed or expanded pluripotency. These advances have largely been based on adaptations of conditions for primed mouse and primate PSCs (Brons et al., 2007; Kishimoto et al., 2021; Tesar et al., 2007; Tsakiridis et al., 2014), involving various combinations of activin A (or TGFβ), FGF, modulators of the Wnt pathway and serum replacement (Alberio et al., 2010; Bogliotti et al., 2018; Choi et al., 2019; Gao et al., 2019; Vilarino et al., 2020). Establishment of PSC cultures has relied on feeder cells in all cases, although a recent paper reported post-derivation expansion of bovine PSCs without feeders (Soto et al., 2021). These developments are encouraging. However, the culture conditions used differ between studies and are not defined, which obfuscates comparisons. Thus, the relatedness of the cultured stem cell lines to the embryo and to one another are unclear. Here, we have investigated derivation of PSCs from pig, sheep and cattle embryos in defined conditions using an identical combination of activin A, FGF2 and the tankyrase inhibitor XAV939, without feeders or serum substitutes.

## RESULTS

### Derivation of pluripotent stem cell cultures from porcine bilaminar disc epiblast

In livestock embryos, the epiblast undergoes formative transition (Smith, 2017) to generate an epithelial embryonic disc prior to implantation (Alberio et al., 2021; Sheng, 2015). We used pig embryos to access the embryonic disc at the pre-gastrulation spherical blastocyst stage. Embryos were collected on embryonic day 11 (E11) from synchronized inseminated sows. We manually dissected out the bilaminar disc and peeled away the underlying hypoblast layer. Single epiblasts were plated intact in four-well plates in N2B27 medium (Mulas et al., 2019) without feeders, serum or serum substitutes on plates coated with a combination of laminin and fibronectin (Fig. S1A). N2B27 was supplemented throughout with activin A (20 ng/ml), FGF2 (12.5 ng/ml) and XAV939 (2 µM), collectively termed AFX. XAV939 is a tankyrase inhibitor that blocks canonical Wnt signalling by stabilising the β-catenin destruction complex (Huang et al., 2009). Cultures were maintained in 5% O_2_ at 38.5°C, the body temperature of pigs (Dukes, 2015).

**Fig. 1.**
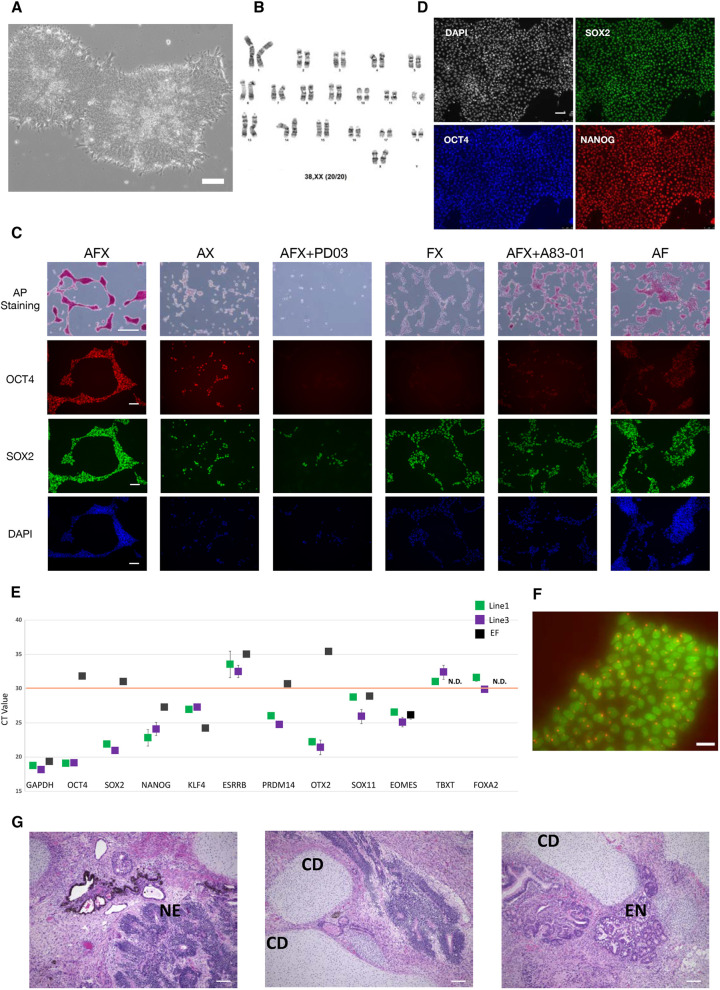
**Derivation of self-renewing pig pluripotent stem cell lines.** (A) Bright-field image of pig stem cells in AFX at P15. Scale bar: 100 µm. (B) G-banding analysis of female line at P21. (C) Cells cultured in the indicated medium for one passage (4 days in total) and assessed by AP staining, and by OCT4 and SOX2 immunostaining. Concentrations of factors were 20 ng/ml activin A, 12.5 ng/ml FGF2, 2 µM XAV939, 2 µM IWP-2, 1 µM PD0325901 and 1 µM A83-01. Scale bars: 500 µm (AP staining); 100 µm (immunostaining). (D) Immunofluorescence staining for OCT4, SOX2 and NANOG. Scale bar: 75 µm. (E) CT values from qRT-PCR analysis of pluripotent and formative/primed gene expression patterns. Orange line marks CT value of 30. EF, pig embryonic fibroblast. TBXT and FOXA2 were not detected (N.D.) in EF. Data are mean±s.d. from technical triplicates. (F) Immunofluorescence staining for H3K27me3 (red) and OCT4 (green) in female AFX line. Scale bar: 25 µm. (G) Teratomas sectioned and stained with Haematoxylin and Eosin. Scale bars: 100 µm. CD, chondrocytes; EN, endoderm epithelium; NE, neuroepithelium.

Epiblasts outgrew and proliferated as flattened epithelial-like monolayers (Fig. S1B). After 6-8 days, we used accutase to dissociate each explant into small clumps that were replated in AFX medium supplemented with a Rho-associated kinase (Rock) inhibitor, Y27632 (10 µM) (Watanabe et al., 2007). Several colonies typically expanded from each original epiblast and at the next passage were transferred together into one well of a 12-well or six-well plate. Thereafter, cultures were routinely passaged every 2-3 days at a ratio of 1/10-1/20. Y27632 was added at passaging and removed after 24 or 48 h. Cells grew as dense monolayers with clear colony borders and little morphological differentiation (Fig. 1A). We plated 10 epiblasts and in each case obtained a continuous stem cell line (expanded for more than 10 passages) (Fig. S1A). Metaphase analysis showed a euploid count of 38 chromosomes (20/20) for three lines examined at passages 8 (two lines) or 21 (Fig. 1B). We also derived lines on MEF feeders and found that they could readily adapt to feeder-free culture in AFX (Fig. S1C). As for primary epiblast, established lines did not attach well to plates coated with fibronectin only, but required fibronectin and laminin.

We investigated the requirement for individual components of AFX and found that all three were required to support continuous expansion of alkaline phosphatase and OCT4-positive cells (Fig. 1C). Furthermore, inhibition of MEK/ERK signalling with PD0325901 caused complete differentiation or death within one passage, whereas blockade of activin/TGFβ receptor signalling with A83-01 caused differentiation with reduced proliferation, although some OCT4-positive cells persisted. In contrast, without XAV939, cells retained alkaline phosphatase but downregulated OCT4 and partially lost SOX2. To test whether the effect of XAV939 is mediated via blockade of Wnt signalling we tested a different mode of suppressing the pathway using IWP2, which prevents Wnt production by inhibiting essential post-translational modification by porcupine (Chen et al., 2009). We found that IWP2 could replace XAV939 and maintain the expansion of alkaline phosphatase and OCT4/SOX2-positive cells (Fig. S1D).

Immunostaining (Fig. 1D) and qRT-PCR (Fig. 1E) showed the presence of OCT4, SOX2 and NANOG transcription factors in expanded porcine AFX cells. Markers of porcine embryonic disc stage, *PRDM14*, *OTX2* and *SOX11*, were also expressed, whereas transcripts for *KLF4* and *ESRRB* that are present in ICM but downregulated in embryonic disc (Ramos-Ibeas et al., 2019) were expressed at very low levels or absent (Fig. 1E). *TBXT* and *FOXA2* transcripts, typically found in primed pluripotent stem cells, were also not significantly expressed. We did detect low expression of *EOMES*, as seen in formative stem cells (Kinoshita et al., 2021). We carried out immunostaining of female cells for the chromatin modification histone-3 lysine 27 trimethylation (H3K27me3), which decorates the inactive X chromosome in female cells (Plath et al., 2003; Silva et al., 2003). The staining showed a single focus of intense signal in each cell (Fig. 1F).

We investigated stable transfection of AFX cells. We introduced a constitutive mKO2 fluorescent reporter transgene using piggyBac transposase-based random integration followed by puromycin selection (1.0 µg/ml). Stably fluorescent cells were readily obtained. We injected reporter cells under the sub-renal and testis capsules of NOD/SCID mice to assess multilineage differentiation potential. At both sites, large mKO2-positive teratomas formed (Fig. S1E,F). In histological sections, we observed various differentiated tissues, including neuroepithelium, pigmented epithelium, cartilage and exocrine epithelium containing secretory vacuoles, indicative of derivatives of all three primary germ layers (Fig. 1G). Together, these findings indicate that AFX comprises the necessary and sufficient signalling environment for derivation and expansion of pluripotent porcine stem cells from embryonic disc stage epiblast.

### Establishment of pluripotent stem cells from ovine and bovine embryos

Encouraged by these findings, we extended the approach to sheep. We similarly dissected embryonic disc stage epiblasts from *in vivo* embryos (E8 and E11) and cultured them in AFX without feeders at 38.5°C. Compared with porcine, we observed that ovine epiblast explants were more liable to overgrowth by differentiated hypoblast-like cells during initial expansion. We therefore avoided bulk passaging in favour of manually picking undifferentiated regions for the first two passages. In this way, we derived eight continuous stem cell lines from 15 embryos. Established ovine AFX stem cells were similar in appearance to porcine, although colonies appeared less compact.

Ovine whole-embryo culture does not progress reliably to the spherical blastocyst stage. However, post-implantation stage PSCs have been derived via *in vitro* development of naïve epiblast in ICM explant cultures in mouse and human (Najm et al., 2011; O'Leary et al., 2012). We therefore isolated sheep ICMs by immunosurgery from E6 and E7 *in vivo* blastocysts and cultured them intact in AFX. After subsequent passaging, we failed to derive stem cell cultures initiated from E6 ICMs, but three out of eight E7 ICMs yielded stable stem cell lines that were morphologically indistinguishable from embryonic disc-derived cultures. The supply of *in vivo* embryos is limiting because only one or two can be obtained per ewe. Therefore, we investigated derivation from *in vitro*-produced blastocysts (German et al., 2015). Following the same ICM outgrowth procedure as for *in vivo* blastocysts, we established five stem cell lines from 13 E7 embryos. Regardless of *in vivo* or *in vitro* origin, ICM explants often became dominated by rapidly expanding differentiated derivatives (Fig. S2A). Careful manual isolation of the undifferentiated area with minimal carry over of differentiated cells was necessary to establish stem cell lines.

Derivations of ovine lines are summarized in Fig. S2B. Immunostaining for H3K27me3 (Fig. S2C) showed a single strong focus in each nucleus of female cells, consistent with an inactive X chromosome. We tested teratoma formation in NOD/SCID mice and obtained multilineage differentiated tumours from both lines tested (Fig. S2D).

Successful derivation from sheep IVF blastocysts prompted us to apply the same approach in cattle. Bovine blastocysts fail to develop beyond day 7 in conventional embryo culture medium. However, we previously showed that N2B27 medium supports development to the spherical blastocyst stage with a larger and more advanced ICM (Canizo et al., 2019; Sandra et al., 2017). Here, we used N2B27 with addition of activin A for 24 h from E8 to E9. We isolated ICMs by immunosurgery from embryos at E7, E8 and E9 (Fig. S2E). Cultured explants were plated in AFX medium as for ovine cell line derivation. Explants from E8 and E9 embryos typically exhibited an embryonic disc-like structure surrounded by differentiated cells (Fig. S2F). An embryonic disc also became, apparent in some, but not all, of the E7 ICM explants. We established 15 cell lines from 27 blastocysts, summarized in Fig. S2G. Similar to the results in sheep, stem cell derivation was least efficient from early ICMs (Fig. S2G). We also injected bovine cells into NOD/SCID mice and obtained tumours with areas of primitive differentiation (Fig. S2H).

**Fig. 2.**
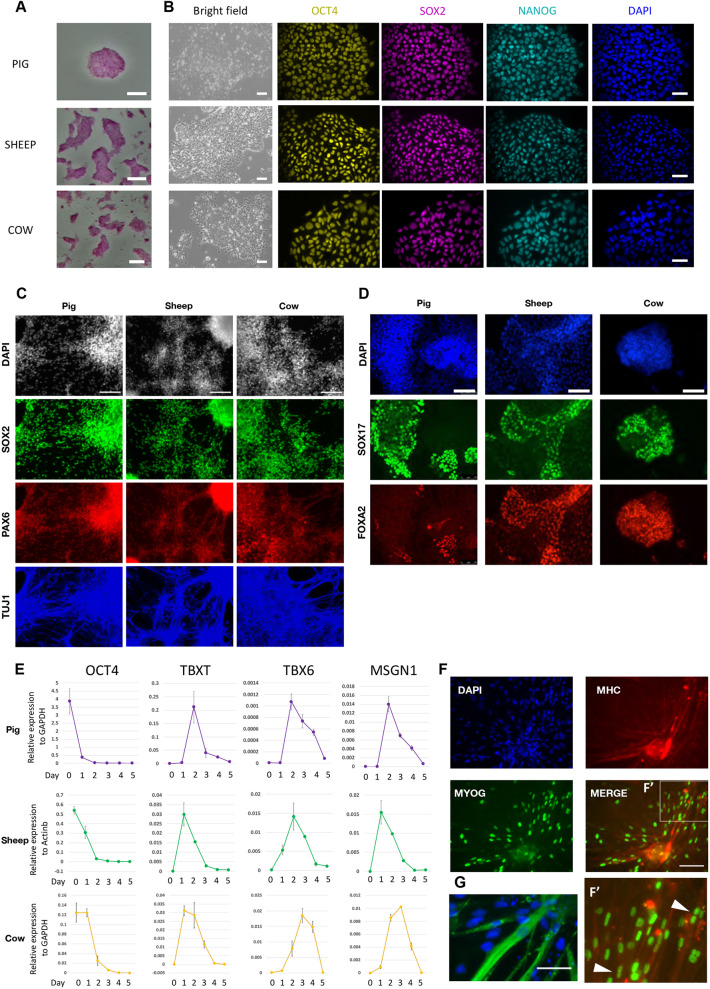
**Establishment of pluripotent stem cells from livestock mammals.** (A) Alkaline phosphatase staining of AFX cell colonies. Passage numbers are: 17 (pig), 20 (sheep) and 12 (cow). AP staining (A) and immunofluorescence of OCT4, SOX2 and NANOG (B) was performed on at least three lines from each species. (B) Bright-field images of EDSCs from pig (P9), sheep (P8) and cow (P21) embryos. Scale bars: 100 µm. Colonies were stained for OCT4, SOX2 and NANOG, and with DAPI. (C) AFX cells differentiated into neural lineage and stained for SOX2 (green), PAX6 (red) and TUJ1 (blue). DAPI images are in grey. Scale bars: 100 µm. (D) AFX cells differentiated into definitive endoderm and stained for SOX17 (green) and FOXA2 (red). DAPI is in blue. Scale bars: 100 µm. (E) qRT-PCR analysis during paraxial mesoderm differentiation. Data are mean±s.d. from technical duplicates. (F) Immunofluorescence staining of MYOG and myosin heavy chain (MHC) in differentiated bovine EDSCs. F′ is a higher magnification of the boxed area with arrowheads indicating multiple nuclei in MHC-positive cells. Scale bar: 100 µm. (G) Differentiated bovine AFX cells immunostained for the striated muscle marker TITIN (green) and DAPI (blue). Scale bar: 25 µm.

Alkaline phosphatase was expressed by porcine, ovine and bovine AFX stem cells (Fig. 2A). By immunostaining, we detected the presence of OCT4, SOX2 and NANOG in almost all cells similarly in lines from the three species (Fig. 2B). Cell lines from each species could readily be expanded for more than 30 passages with no change in morphology. In ovine and bovine cultures, as for porcine, XAV939 could be replaced by IWP2 (2 µM) with no detriment to expansion or pluripotency factor expression over several passages (Fig. S2I).

We adopted protocols commonly used for human PSCs to investigate *in vitro* differentiation of AFX cells. For neural induction, we applied dual SMAD inhibition (Chambers et al., 2009), passaging cultures at an intermediate time point as they reached confluence. From 14 days we began to observe networks of extended cellular processes and detected abundant expression of the neural lineage markers PAX6 and SOX2, and of the early neuronal marker type III β-tubulin (TuJ1) in all three species (Fig. 2C). For definitive endoderm, we simplified a protocol for human PSCs (Loh et al., 2014), treating cells with activin A plus the GSK3 inhibitor CH99021 for 24 h, then with activin A only for 2 days. We obtained SOX17 and FOXA2 double-positive endoderm cells from each species (Fig. 2D). For mesoderm, we adapted a protocol for stepwise induction of pre-somitic mesoderm and paraxial mesoderm (Chal et al., 2016). qRT-PCR analysis showed upregulation of paraxial mesoderm lineage markers in all three species (Fig. 2E). We investigated potential for further differentiation of bovine cells along the myogenic lineage, a prerequisite for biomanufacturing cellular meat products. Paraxial mesoderm populations were treated as described for human PSC differentiation (Chal et al., 2016) and maintained for 6 weeks in the presence of IGF and HGF. We detected patches of cells that co-stained for MYOG and myosin heavy chain (Fig. 2F). Immunostaining for TITIN showed striations indicative of skeletal muscle differentiation (Fig. 2G).

### Transcriptome profiling of AFX cell identity

**Fig. 3.**
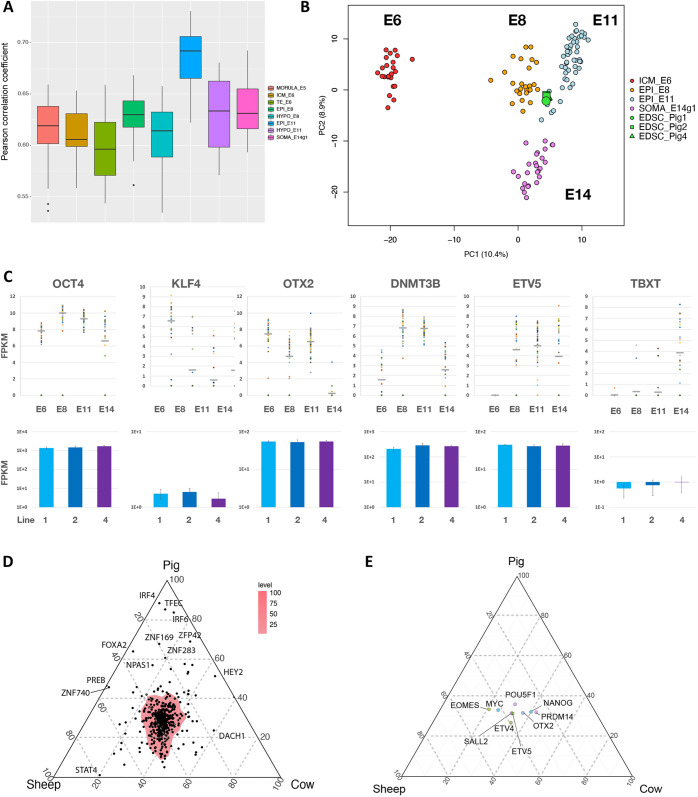
**Transcriptome analysis of embryonic disc stem cells.** (A) Pearson correlation of pig EDSC transcriptome with porcine embryo stages (Ramos-Ibeas et al., 2019; Zhu et al., 2021). For E14, we used the g1 population of posterior cells that are mostly OCT4 positive. The box extends from the lower to the upper quartile and the horizontal line is the median. Whiskers extend to 1.5 times the interquartile range. (B) Projection of porcine EDSCs on PCA of porcine embryo stages computed using the top 1000 variable genes. (C) Expression values of selected marker genes in porcine and EDSCs. Upper: embryo scRNAseq FPKM values with median marked by horizontal bar. Lower: cell line RNAseq log FPKM values. Error bars represent s.d. from triplicate samples. (D) Ternary plot for EDSCs of the three species computed for 555 orthologous transcription factor genes expressed in porcine E11 epiblast. The region of highest density of shared factors is shaded. Differentially expressed genes are indicated. (E) Ternary plot as in D, with selected pluripotency-associated factors labelled.

To examine whole-transcriptome features of AFX stem cells, we prepared RNAseq libraries in triplicate from three porcine lines, one male and two female, between passages 6 and 10. We compared the cell line transcriptomes with our published RNAseq data from stages of porcine embryo development from morula (E4) to gastrulation (E14) (Ramos-Ibeas et al., 2019; Zhu et al., 2021). Pearson correlation showed highest global similarity to embryonic disc (E11) (Fig. 3A, Fig. S3A). Principal component analysis (PCA) also indicated relatedness to pluripotent embryonic disc and separation from both earlier epiblast and gastrulating cells (Fig. 3B). Fig. 3C shows normalized expression values for selected markers in embryo stages and three porcine stem cell lines. AFX cells display factors enriched in the embryonic disc (*OTX2*, *DNMT3B* and *ETV5*) and have little expression or expression of both the early epiblast (naïve pluripotency) factor *KLF4* and the gastrulation marker *TBXT*. Therefore, we accorded AFX cells the title embryonic disc stem cells (EDSCs).

We then undertook a comparison of porcine EDSCs with our ovine and bovine cell lines. Global analysis is confounded by the current limitations of gene annotation in these animals. We therefore focused on orthologous transcription factors highly expressed in the porcine embryonic disc (E11) (Ramos-Ibeas et al., 2019). Ternary plots computed for this group of 555 genes showed the vast majority were present in EDSCs of each species and with similar relative expression indicated by the high-density area in the centre of the plot (Fig. 3D). Recognised core and formative pluripotency factors were contained in the high-density region (Fig. 3E). We conclude that, for all three species, AFX cell lines display a similar transcription factor expression profile and may be considered as EDSCs. We also saw that *de novo* methyltransferases DNMT3A and DNMT3B, which are upregulated during formative transition (Fig. 3C) (Smith, 2017), were expressed in EDSCs of each species (Fig. S3B). Of note, ovine samples included both embryonic disc and ICM-derived cell lines and bovine lines were derived from late ICM explants. Thus, the AFX culture environment consistently captures embryonic disc stage cells from epiblast progression *in vitro*. This is consistent with the culture condition determining the stem cell state that is captured, as previously shown for derivations of primed PSCs corresponding to late epiblast starting from mouse and human ICMs (Najm et al., 2011; O'Leary et al., 2012).

We used live cell staining and flow cytometry to investigate expression of cell surface markers found on human PSCs. Among the antibodies tested, only SSEA4 showed high expression of in all three species (Fig. S3C). The other markers examined were either weakly positive (CD57 and CD90 in pig only) or negative (SSEA1, TRA1-60, TRA1-81 and CD24). However, CD24 and PODXL1 (recognized by TRA1-60 and TRA1-81) are expressed in porcine RNA-seq data (Fig. S3D). Therefore, at least in those cases, the antibodies may have no or poor species cross-reactivity.

We also compared EDSCs with other recently reported pluripotent stem cell cultures from livestock animals. Two studies have described porcine stem cell propagation. Choi et al. used a combination of activin A, FGF, GSK3 inhibition and tankyrase inhibition, together with KSR and feeders (Choi et al., 2019). Gao et al. derived so-called expanded potential stem cells in medium containing a GSK3 inhibitor, a Src inhibitor, a tankyrase inhibitor, vitamin C, LIF, activin A and serum on feeders (Gao et al., 2019). We generated ternary plots as above using published transcriptome data from those studies. Expression of the set of porcine E11 epiblast transcription factors was similar between EDSCs and cells of Choi et al. but less so with expanded potential stem cells (Fig. S3E). PCA computed using all expressed genes confirmed similarity between EDSCs and the cell lines of Choi et al. and distinction from expanded potential stem cells (Fig. S3F).

A medium termed CTFR, containing FGF and the tankyrase inhibitor IWR1, has been used in combination with feeders to derive pluripotent cell lines from sheep and cattle ICMs (Bogliotti et al., 2018; Vilarino et al., 2020). We compared sheep EDSC transcriptomes with available data for two lines of sheep CTFR cells and saw very similar expression of embryonic disc transcription factors (Fig. S3G). Notably, however, NANOG transcript levels were lower in the CTFR cells. This is consistent with the reported absence of NANOG immunostaining in ovine CTFR cells (Vilarino et al., 2020), in contrast to ready detection in AFX cells (Fig. 2A). Bovine CTFR cells also expressed embryonic disc-enriched transcription factors (Fig. S3H).

Overall, these transcriptome analyses indicate a high degree of overlap between EDSCs of pig, sheep and cattle, with relatedness to the porcine E11 embryonic disc and other recently described livestock pluripotent stem cells but less so with expanded potential stem cells.

### Targeted genetic manipulation and nuclear transfer

**Fig. 4.**
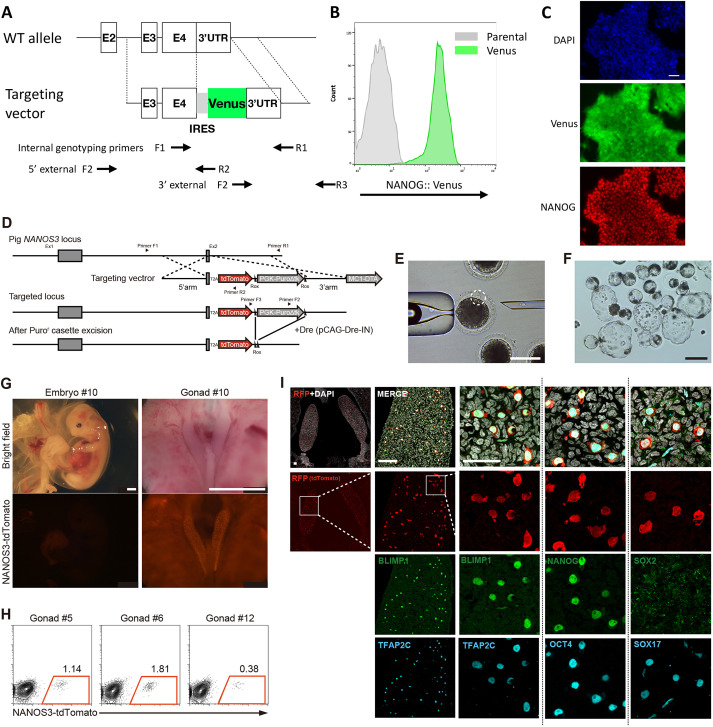
**Targeting and nuclear transfer.** (A) Design of the *NANOG* targeting vector. (B) Flow cytometry analysis of *NANOG*::Venus knock-in line. (C) Image of Venus fluorescence and Nanog immunostaining from the same line as in B. Scale bar: 50 µm. (D) Design of *NANOS3* targeting vector. (E) Injection of single EDSC into the perivitelline space of an enucleated oocyte. Dashed circle highlights EDSC. Scale bar: 100 µm. (F) Cloned embryo development to blastocyst stage *in vitro*. Scale bar: 200 µm. (G) Cloned embryo retrieved on E29 with tdTomato expression in embryonic gonads. Scale bars: 2.5 mm. (H) Flow analysis of tdTomato expression in gonads from three independent cloned embryos. (I) Immunostaining of sectioned gonad for tdTomato and indicated transcription factors. SOX2 staining was not detected. Scale bars: 100 µm.

To assess the suitability of EDSCs for genome engineering, we undertook CRISPR/Cas9-mediated gene targeting. We first designed vectors for insertion of reporters into the *NANOG* gene in porcine EDSCs (Fig. 4A). Two lines of EDSCs were co-transfected with CRISPR/Cas9 gRNA and either mKO2 or Venus targeting constructs. After transfection, we passaged cells twice before single cell sorting for reporter-positive cells. In trials with the mKO2 construct, efficiencies of clonal expansion were 21.9±3.1% and 31.6±12.1% (*n*=2), respectively, for the two lines. In a repeat experiment using the brighter Venus reporter, we confirmed targeting by genomic PCR (gPCR) (Fig. S4A) and validated expression by flow cytometry and imaging (Fig. 4B,C). We similarly tested genetic modification and clonal expansion in ovine EDSCs, targeting *DPPA3*. Transfected cells were plated at low density and selected with 1.0 µg/ml of puromycin. Targeted clones identified by PCR genotyping were expanded and transiently co-transfected with Cre and GFP expression vectors. After single cell sorting, subclones were re-genotyped by PCR (Fig. S4C). Metaphase counts of two independent targeted clones showed 90% and 74% diploid cells (54 chromosomes) (Fig. S4D). Last, we examined genetic modification and clonal expansion of bovine EDSCs. We introduced a constitutive GFP expression vector by electroporation and selected stable transfectants in puromycin. After clonal plating, 10 colonies were picked and expanded for 2 weeks. We prepared metaphase spreads from two of these clones and counted 69% and 33% diploid cells (60 chromosomes).

We then investigated the potential for creating genetically modified animals. For this, we chose to introduce a tdTomato reporter into the germline-specific gene *NANOS3* in porcine EDSCs (Fig. 4D). After transfection followed by puromycin selection, three out of 18 male clones and two out of 18 female clones were identified as correctly targeted by gPCR (Fig. S4B). The selection cassette was then removed by transient transfection with Dre recombinase (Anastassiadis et al., 2009). Excision was confirmed by gPCR on expanded clones (Fig. S4B). We prepared metaphase spreads from a male and a female targeted line. Both were karyotypically normal (Fig. S4G). We used the *NANOS3*-tdTomato porcine EDSCs as donors for nuclear transfer (NT). After injection and electrofusion of EDSCs with enucleated oocytes, a proportion of embryos developed into morphologically normal blastocysts (Fig. 4F, Fig. S4H). The efficiency appeared higher for the male line (29%) than the female line (15%) (Fig. S4D). Therefore, we chose the male line to pursue NT embryo development *in vivo*. From 210 oocytes electrofused with EDSCs, we obtained 58 blastocysts (27.8%) (Fig. 4E,F, Fig. S4D). After uterine transfer to recipients, we recovered five morphologically normal foetuses at E29 (Fig. S4D). By this stage the body plan has been laid down and major organs are forming. We observed tdTomato expression restricted to the gonads, as expected for primordial germ cells (PGCs) by this stage (Fig. 4G). Flow cytometry analysis substantiated the presence of cells expressing tdTomato in dissociated gonadal tissue from three different embryos (Fig. 4H). Immunostaining showed that the NANOS3-tdTomato-positive cells expressed germ cell markers, BLIMP1, TFAP2C, OCT4 and NANOG, confirming faithful labelling of pig PGCs by the reporter. Of note, *NANOS3*-tdTomato-positive pig PGCs also expressed SOX17 but lacked SOX2, shared features of pig and human PGCs that differ from mouse (Kobayashi et al., 2017) (Fig. 4I). These results demonstrate the fidelity of the knock-in reporter and confirm the origin of the foetuses from the genetically manipulated EDSCs.

## DISCUSSION

Our findings demonstrate that identical, well-defined, and relatively simple culture conditions support derivation of PSCs related to the embryonic disc for the three major livestock animals: pig, sheep and cattle. Under stimulation with activin A and FGF, together with inhibition of the Wnt pathway, EDSCs can be expanded continuously without feeder cells, serum or serum replacement. They retain a global transcriptome signature of bilaminar disc epiblast. Accordingly, they do not express factors specific to naïve pluripotency and also largely lack lineage-affiliated gene expression that is characteristic of gastrulation stage epiblast and primed mouse PSCs (Kojima et al., 2014; Osteil et al., 2019). EDSCs differentiate in teratomas and into multiple lineages *in vitro*, including skeletal muscle. They can readily be genetically manipulated, clonally expanded and used as donors for nuclear transfer. The establishment of stable stem cell lines from different species in a delimited signalling environment will facilitate gene regulatory network comparisons and elucidation of the relationship between *in vitro* cell lines and stages of pluripotency in the embryo. Furthermore, robust, standardised and scalable PSC culture will be advantageous for genome engineering in these species and of paramount importance for the emerging field of cellular agriculture and the promise of sustainable meat production (Post et al., 2020).

Interestingly, feeder and serum-free AFX conditions similar to those employed here for EDSCs have been used to propagate mouse EpiSCs (Kojima et al., 2014; Osteil et al., 2019; Tsakiridis et al., 2014) and conventional human PSCs (Rostovskaya et al., 2019). Livestock pluripotent stem cells have recently been established on feeders in medium with overlapping components. FGF2 has been used with an alternative tankyrase inhibitor, IWR-1, in both cattle and sheep (Bogliotti et al., 2018; Vilarino et al., 2020). FGF2, IWR-1 and activin A plus GSK3 inhibition, and knockout serum replacement were employed to derive and propagate pig PSCs (Choi et al., 2019). We observed related gene expression between EDSCs and cells from both of those feeder-dependent protocols. We surmise that those formulations support expansion of similar EDSCs. Indeed, a recent report showed that bovine PSCs derived on feeders in FGF and IWR-1 could be adapted to feeder-free culture by addition of activin (Soto et al., 2021), a signalling environment that is likely to be functionally equivalent to AFX. In contrast, expanded potential stem cells, which are purported to represent an early embryonic stage (Gao et al., 2019; Zhao et al., 2021), are transcriptomically distinct from EDSCs.

We found that EDSCs can differentiate into somatic germ layers *in vitro* in response to protocols developed for human PSCs. Myogenic differentiation from EDSCs offers a starting point for the replacement of animals in the generation of meat products (Rubio et al., 2020). Optimisation and generation of other key lineages, such as adipose tissue, will be required along with bioreactor scale-up, but it is already encouraging that myotubes can be detected without genetic manipulation. It may be anticipated that EDSCs can be derived by somatic cell reprogramming, which would enable their generation from elite livestock specimens.

EDSCs are readily amenable to genetic modification and our results demonstrated retention of a normal karyotype after two rounds of clonal propagation required for gene targeting and marker excision. The targeted porcine EDSCs displayed competence to support foetal development by nuclear transfer. Although somatic cell nuclear cloning is well established in pigs, previous efforts using putative pluripotent cells have not yielded successful embryo development *in vivo*. Fan et al. reported a large nuclear transfer study using porcine iPSCs and concluded that persistent activity of reprogramming transgenes compromised embryonic development (Fan et al., 2013). Only after *in vitro* differentiation and associated silencing of transgenes did they obtain a low frequency of full-term development. Our findings indicate that there is no intrinsic barrier to cloning from pluripotent pig stem cells. Compared with fibroblasts, long-term proliferation and clonal expansion, as displayed by EDSCs, are highly advantageous for advanced genome engineering. With protocol development to increase nuclear transfer efficiency similar to that obtained with fibroblasts, use of EDSCs should facilitate complex genetic enhancement of livestock and generation of large animal models of human disease. A further potential application is in creation of genetically compromised animal hosts with niches for production of human tissues and organs (Rashid et al., 2014).

Recently, we described derivation of formative pluripotent stem (FS) cells in mouse and human (Kinoshita et al., 2021). Their propagation relies on activin A and XAV939 but unlike EDSCs does not require exogenous FGF. However, FS cells are dependent on MEK/ERK signalling, likely activated by autocrine FGF (Kinoshita et al., 2021). In future studies, it will be informative to examine the relatedness of EDSCs to FS cells, in terms of transcriptome features, chromatin organization and functional attributes of chimaera colonization and germ cell formation. Positioning of EDSCs on the formative to primed pluripotency trajectory will also benefit from greater temporal resolution of mid- to late-epiblast transcriptome progression in embryos of the different species. The ability to derive and propagate similar pluripotent stem cells from species that are phylogenetically distant (pigs versus sheep/cattle=64 million years, sheep versus cattle=25 million years) by applying a common signalling environment is suggestive of a conserved attractor state (Enver et al., 2009) during mammalian epiblast progression (Smith, 2017). Defining the gene regulatory network in EDSCs of different species will reveal the extent to which they represent equivalent cell states.

In summary, these findings establish a defined culture condition for capturing what may be a common pluripotent stem cell state from diverse livestock mammals. EDSCs provide a new opportunity for comparative mammalian embryology, enhanced potential for animal genetic engineering and a sustainable raw material for cellular agriculture.

## MATERIALS AND METHODS

### Animal studies

Procedures involving animals in the UK have been approved by the School of Biosciences Ethics Review Committee (16/000099), University of Nottingham, and were carried out under authority of UK Home Office project licence P13302F08. Experiments using porcine embryos in Japan were performed in accordance with the animal care and use committee guidelines of the National Institute for Physiological Sciences and Meiji University. Teratoma experiments were performed under guidelines of the Institutional Animal Care and Use Committee of the Institute of Medical Science, University of Tokyo, Japan.

### EDSC derivation

#### Porcine

Pig E9-E11 embryos were produced by artificial insemination of synchronized sows. Embryos were collected by flushing the uterus with PBS supplemented with 1% foetal calf serum (FCS). Epiblasts from the embryonic disc stage were manually dissected under a stereomicroscope. Epiblasts were transferred onto laminin- (10 µg/ml L2020, Merck or L511-E8, Amsbio) and fibronectin- (16.7 µg/ml) coated four-well plates. Outgrowths were dissociated with Accutase and transferred to a freshly prepared four-well plate in the presence of 10 µM of a Rho-associated kinase inhibitor, Y27632.

#### Ovine

*In vivo* E6-E11 sheep embryos were obtained following insemination and collected from the uteri by flushing with PBS containing 1% FCS. Embryonic disc stage epiblasts were manually dissected under a stereo-microscope and plated on coated four-well plates as described for porcine cells. For *in vitro* ovine embryo production (German et al., 2015), ovaries were collected from a local slaughter house, transported to the laboratory within 3 h in warm PBS (30-35°C) and oocytes retrieved. ICMs were isolated by immunosurgery at E7. Isolated ICMs were plated intact on coated four-well plates as above. Spontaneously differentiated cells were manually removed by mouth pipette.

#### Bovine

Bovine blastocysts were produced *in vitro* as previously described (Alberio et al., 2000). At E7, embryos were transferred to N2B27 and from E8 they were supplemented with 20 ng/ml activin A. Immunosurgery and ICM plating were carried out on E7-E9 embryos as for sheep.

### EDSC maintenance

Porcine, ovine and bovine EDSCs were derived and maintained in AFX medium consisting of 20 ng/ml activin A, 12.5 ng/ml FGF2 and 2 µM XAV939 in N2B27 medium (Nichols and Ying, 2006). Cells were maintained on laminin- (10 µg/ml) and fibronectin- (16.7 µg/ml) coated plates. Accutase was used for dissociation and cells were collected and pelleted in DMEM/F12 supplemented with 0.03% BSA. Y27632 was added when passaging porcine EDSCs but was not routinely used for ovine or bovine lines. Cell cultures were periodically screened for mycoplasma by PCR assay and tested negative.

### Teratoma formation

Porcine EDSCs in one well of a six-well plate were transfected with 1.6 µg of pPBCAG-mKO2-IP and 0.4 µg of pCAG-PBase using TransIT LT-1 (Mirus) and selected with 1.0 µg/ml of puromycin. Transfected porcine EDSCs or unlabelled ovine EDSCs were injected into kidney capsules or testes of NOD/SCID mice at ∼5×10^5^ cells per site. Animals were sacrificed after 4-6 weeks. For bovine EDSCs, 1×10^6^ cells were suspended in ice-cold Matrigel (BD) and injected subcutaneously. Teratomas were collected 8 weeks after transplantation. After fixation, teratomas were embedded in paraffin wax, sectioned and stained with Haematoxylin and Eosin for histological inspection. We tested one line for porcine and bovine, and two lines for ovine EDSCs.

### *In vitro* differentiation

Neural differentiation was performed as described for human conventional PSCs (Chambers et al., 2009). For endoderm differentiation, cells were treated with 20 ng/ml activin A and 3 µM CHIR99021 for the first 24 h and activin A only for the following 48 h. Paraxial mesoderm differentiation was performed as follows. Cells were treated with 3 µM CHIR99021 and 500 nM LDN193189 in DMEM/F12 for the first 24 h and 3 µM CHIR99021, 500 nM LDN193189 and 20 ng/ml FGF2 from day 2 to day 5. Skeletal muscle induction was initiated from day 5 by switching the medium supplement to 15% KSR, 10 ng/ml HGF, 2 ng/ml IGF, 20 ng/ml FGF2 and 500 nM LDN193189 for 2 days, to KSR and IGF for another 2 days, and to HGF and IGF thereafter. At least two independent lines from each species were tested for lineage induction. Skeletal muscle maturation was performed on one bovine line.

### Metaphase chromosome analysis

EDSCs were treated with KaryoMax colcemid (Gibco) for 2.5 h. Cells were collected and resuspended in pre-warmed 0.075 M KCl and incubated for 15 min at room temperature. 100 µl of freshly prepared fixative solution (methanol:glacial acetic acid=3:1) were added into the suspension and cells pelleted. Cells were resuspended in fixative (250-500 µl) and up to 20 µl spread per glass slide. Spreads were stained with DAPI and imaged using a Leica DMI4000. G-banding and karyotype analysis of pig EDSCs was performed by TDL Genetics LTD (UK) and Nihon Gene Research Laboratories (Japan).

### qRT-PCR analysis

Total RNAs were isolated with Reliaprep RNA miniprep kit (Promega). cDNAs were prepared by GoScript reverse transcription system (Promega). PCR was performed using SYBR Green enzyme mix (Thermofisher). The list of primers is in Table S2.

### Immunofluorescence analysis

Cells were fixed with 4% PFA for 15 min at room temperature. Cells were blocked with 5% skimmed milk or BSA in PBS, 0.1% Triton X. Primary and secondary antibodies were incubated for 1 h at room temperature or overnight at 4°C. Images were taken using a Leica DMI4000. Antibodies used are all commercially available (see Table S3).

### Alkaline phosphatase staining

Alkaline phosphatase staining was performed following the manufacturer's instruction (86R-1KT, Sigma Aldrich).

### Gene targeting and stable transfection

#### *NANOG*::Venus knock-in

pEDSCs were transfected with gRNA expression construct (0.8 µg), Cas9 expression construct (0.8 µg) with *NANOG*::Venus knock-in vector (0.4 µg) by TransIT LT-1 (Mirus) in one well of the six-well plate. Cells were cultured and passaged once, and a single cell sort was performed. Expanded clones were genotyped by touch-down PCR (denature at 98°C for 10 s, drop the annealing temperature by 1°C per cycle from 65°C for 15 s for the initial 10 cycles, followed by another 35 cycle of 56°C of annealing temperature, extension at 65°C for 3 min) using LongAmp Taq polymerase (NEB). gRNA sequence and genotype primers are provided in Table S1.

#### *NANOS3*::tdTomato knock-in

Reverse transfection was carried out using lipofectamine 2000 (Thermo Scientific) as described previously (Kobayashi et al., 2017). pEDSCs (1.5×10^5^) were suspended in 100 µl of Opti-MEM (Thermo Fisher Scientific) containing targeting vector (2.0 µg), CRISPR/Cas9 plasmid (1.0 µg) and lipofectamine complex, and then left for 5 min at room temperature. pEDSCs were then seeded onto puromycin-resistant MEF and, 48 h later, 0.8 µg/ml puromycin was added to the culture medium for selection. Colonies were picked and genotyped by PCR. Targeted clones were transfected with Dre expression vectors and expanded from clonal density. Clones were genotyped to detect excision of the puromycin selection cassette. gRNA sequences and genotype primers are provided in Table S1. Genotyping PCR was performed using Tks Gflex DNA Polymerase (Takara).

#### *DPPA3*::mKO2 knock-in

shEDSCs were transfected with a gRNA expression construct (0.8 µg), a Cas9 expression construct (0.8 µg) with DPPA3::Venus knock-in vector (0.4 µg) using TransIT LT-1 (Mirus) in one well of a six-well plate. 1000 cells were replated into 10 cm plate with 10 µg/ml of Rock inhibitor. Clones were picked and genotyped by touch-down PCR (denature at 98°C for 10 s, drop the annealing temperature by 1°C per cycle from 65°C for 15 s for the initial 10 cycles, followed by another 35 cycles of 56°C of annealing temperature and extension at 65°C for 3 min) using LongAmp Taq polymerase (NEB). Expanded targeted clones were co-transfected with pCAG-GS-Cre and pPBCAG-GFP-IP plasmids using transit LT1. GFP-positive cells were isolated by single cell flow sorting into 96-well plates 48 h after transfection. Expanded clones were genotyped by gPCR. gRNA sequence and genotype primers are listed in Table S1.

#### Stable transfection of bovine EDSCs

bEDSCs (1×10^6^) were electroporated with 2 µg of pPBase and 8 µg of pPBCAG-GFP-IP plasmids. Electroporation was performed using NEPA 21 electroporator and EC-002S NEPA Electroporation Cuvettes (2 mm gap) as follows: poring pulse, 115 V, length 2.5 ms, two pulses with 50 ms interval, D.rate 10%, polarity +; transfer pulse, 20 V, length 50 ms, five pulses with 50 ms interval, D.rate 40%, polarity +/−. After electroporation, cells were seeded at 1×10^6^/well in a six-well dish. Puromycin (0.5 µg/ml) selection was started on the next day for 4 days (one passage in between). Clonal expansion was achieved by limiting dilution of a single cell suspension into 96-well TC plates.

### Nuclear transfer

Nuclear transfer of NANOS3::tdTomato knock-in pEDSCs was performed as described for somatic cell nuclear transfer (Kurome et al., 2008; Matsunari et al., 2013) without cell cycle synchronization. In brief, a single EDSC was electrically fused with an enucleated oocyte. The reconstructed embryos were electrically activated and cultured in porcine zygote medium 5 (PZM-5, Research Institute for Functional Peptides, Yamagata, Japan) (Yoshioka et al., 2008) for 3 h in the presence of 5 μg/ml cytochalasin B and 500 nM scriptaid, and embryos were then cultured with 500 nM scriptaid (Zhao et al., 2009) for another 12-15 h. After these treatments, cloned embryos were cultured in PZM-5 in 5% CO_2_, 5% O_2_ and 90% N_2_ at 38.5°C. On day 4, morula stage embryos were transferred in fresh PZM-5 supplemented with 10% FBS. On day 6, blastocysts were surgically transferred into the uterine horns of oestrus-synchronized recipients.

### Flow cytometry and cell sorting

For surface maker analysis, EDSCs were washed with PBS and dissociated with Cell Dissociation buffer, Enzyme Free, Hanks's Balanced Solution (Gibco). Dissociated cells were incubated with fluorophore-conjugated antibody on ice for 20 min and analysed using a BD FACS Fortessa with FlowJo software. DAPI and PI were used to gate out dead cells. Single Venus-positive DAPI- negative pEDSCs transfected with a NANOG-Venus targeting construct and single GFP-positive DAPI-negative shEDSCs were sorted into laminin/fibronectin coated 96-well plates in AFX medium supplemented with 10 µM Rock inhibitor using a BD FACS Fusion instrument. Established *NANOG*::Venus lines were analysed using a BD FACS Fortessa with FlowJo software. For the analysis of porcine PGCs in NANOS3::tdTomato cloned foetuses, the gonads were digested using 0.1% collagenase type IV/PBS for 15 min followed by 0.25% trypsin/EDTA for 5 min. The cells were analysed using an SH800 flow cytometer (SONY) with FlowJo software.

### RNA-sequencing

Cells were lysed in Trizol (Thermo Fisher Scientific) and total RNAs were purified by Purelink RNA mini kit (Thermo Fisher Scientific). Ribosomal RNAs were removed with Ribo-zero rRNA removal kit (Illumina) for porcine samples and Qiaseq FastSelect RNA removal kit (Qiagen) for ovine and bovine. Libraries were prepared using the NEBNext Ultra II Directional RNA Library Prep Kit for Illumina (NEB).

### Data processing

Trim-Galore! v0.6.5 (https://www.bioinformatics.babraham.ac.uk/projects/trim_galore/) was used to trim adapter sequences and remove low-quality base calls from the 3′ end of reads of porcine, ovine and bovine EDSC samples with default parameters. STAR v2.7.3a (Dobin et al., 2013) was used to map trimmed reads to the reference genome assembly of each species (porcine, Sscrofa11.1; ovine, Oar_v3.1; bovine, ARS-UCD1.2). FeatureCounts from SubRead v2.0.0 (Liao et al., 2014) was used to quantify expression to gene loci. We obtained normalised counts from Bogliotti et al. (2018) (GEO accession number GSE110040) while samples from Vilarino et al. (2020) (SRA accession number PRJNA609175) were processed identically to our sheep EDSC samples. Porcine (Gao et al., 2019; Array Express accession number E-MTAB-7253; Choi et al., 2019; GEO accession number GSE120031) samples were trimmed with Trim-Galore! v0.5.0 with a stringency of 6 and aligned to the porcine reference genome using TopHat2 (Kim et al., 2013) with known gene models provided. Gene counts were generated with FeatureCounts from subread 1.5.0.

### Analysis

Analyses were performed on log_2_FPKM normalised values using R (ver.4.0.3) (https://www.r-project.org/). Only protein-coding genes were considered. For interspecies comparison, genes orthologous between pig, cow and sheep were identified using BioMart (Smedley et al., 2015). For porcine genes with multiple orthologous loci between the species (due to one-to-many or many-to-many relationships), a single locus per species was selected according to the similarity scores against the porcine gene. Pig single cells expressing fewer than 4625 genes per sample were excluded from the analysis.

### Principal component analysis

Analyses were performed using the R ‘prcomp’. To compare the pig EDSCs and pig embryo single cell data (accession numbers GSE112380 and GSE155136), the principal components of the pig single cells were computed, using the top 1000 variable genes across the dataset. The pig EDSC samples were then projected onto this PCA space using the ‘predict’ function. Similarly, the principal components for the Gao et al. (2019) and Choi et al. (2019) samples were computed using all expressed genes across the combined datasets, onto which the pig EDSC samples were projected.

### Correlation matrices, boxplots and heatmaps

Correlation matrices and boxplots between porcine EDSC RNAseq data and porcine embryo single cell RNAseq were generated using Pearson's correlation coefficient for all genes.

### Ternary plots

Porcine transcription factors expressed above a mean of 1 FPKM in pig epiblast E11 cells (555 genes) and above a mean of 1 FPKM in at least one species plotted were used to create ternary plots using the R package ‘ggtern’ (Hamilton and Ferry, 2018). Gene expression values were averaged for each dataset then divided by the sum of the averaged values so that the sum for each gene across the three datasets is one. Density areas were computed using 2D kernel density estimation.

## Supplementary Material

Supplementary information

Reviewer comments
