# Supplementary material for: Pluripotent stem cells related to embryonic disc exhibit common self-renewal requirements in diverse livestock species
Source: Development. 2021 Dec 7;148(23):dev199901. doi: 10.1242/dev.199901 (PMC8714072; doi:10.1242/dev.199901)
Supplement: Supplementary information [file develop-148-199901-s1.pdf]

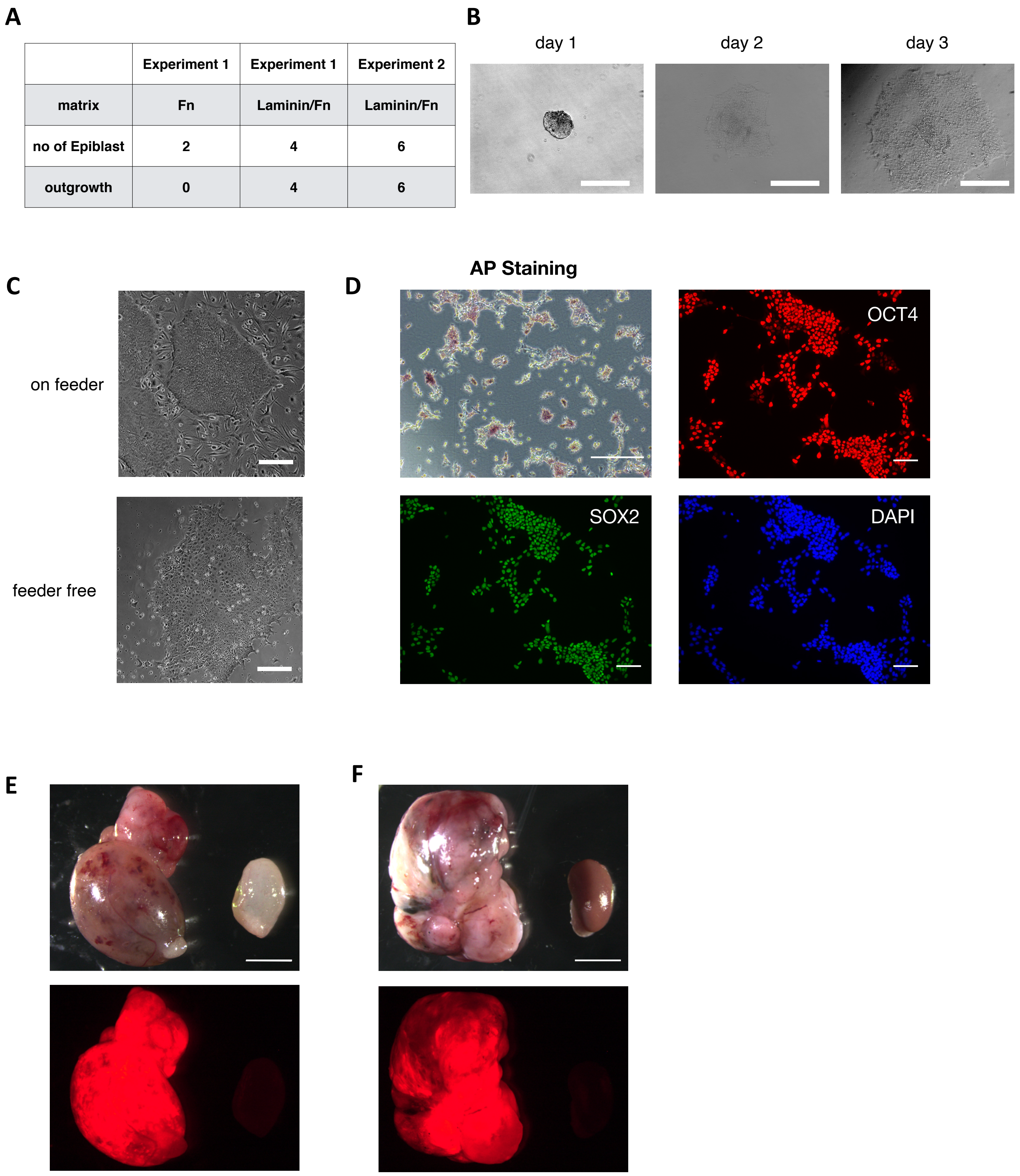

	Experiment 1	Experiment 1	Experiment 2
matrix	Fn	Laminin/Fn	Laminin/Fn
no of Epiblast	2	4	6
outgrowth	0	4	6

Fig. S1. Related to Fig. 1.

(A) Summary of derivation from E11 pig embryos. (B) E11 epiblast outgrowth over the initial three days after plating. Scale bars, 500 µm. (C) Higher magnification images of pig AFX cells. Epiblast-derived cultures established on feeders adapt to culture in AFX without feeders within 5 passages. Scale bar, 200 µm. (D) Pig AFX cells cultured in porcupine inhibitor IWP-2 instead of tankyrase inhibitor XAV939 for one passage (4 days in total), as in Fig. 1C. Scale bars: AP staining, 500 µm; immunofluorescence images, 100 µm. (E, F) Teratomas generated from pig AFX cells in testis (E) and kidney capsule (F). Scale bars, 5mm.

Development • Supplementary information

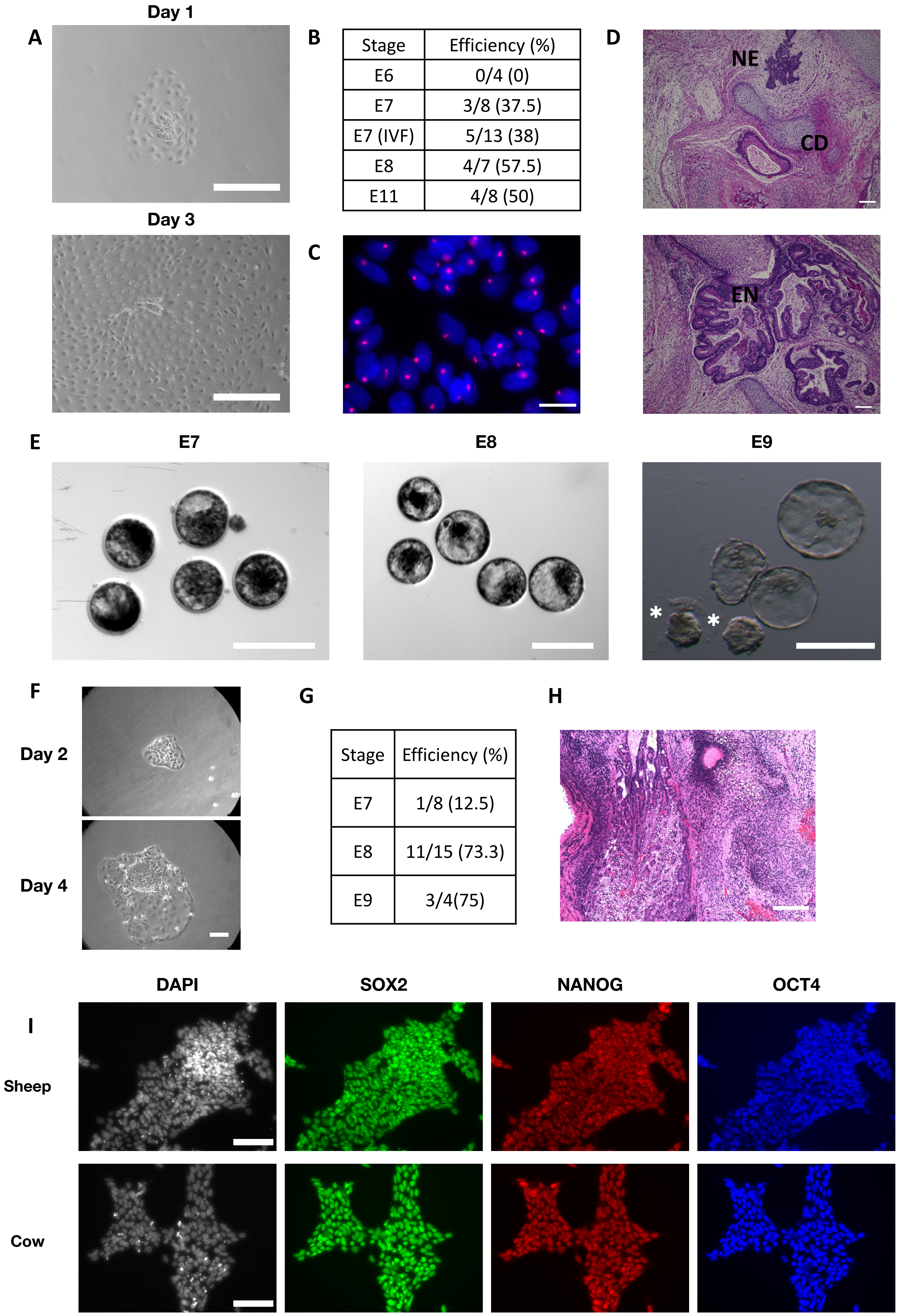

B

Stage	Efficiency (%)
E6	0/4 (0)
E7	3/8 (37.5)
E7 (IVF)	5/13 (38)
E8	4/7 (57.5)
E11	4/8 (50)

G

Stage	Efficiency (%)
E7	1/8 (12.5)
E8	11/15 (73.3)
E9	3/4(75)

Fig. S2. Related to Fig. 2

(A) Sheep ICM outgrowth. Cultures were dominated by differentiated cells by day 3. Scale bars, 250 µm. (B) Summary of sheep AFX cell derivation from different developmental stages. (C) H3K27me3 staining (red) of female sheep AFX cells. DAPI in blue. Scale bar, 25 µm. (D) Teratomas from sheep AFX cells sectioned and stained with hematoxylin and eosin. Scale bars, 100µm. NE; neuroepithelium, CD; chondrocytes EN; Endoderm epithelium (E) Morphology of in vitro developed bovine blastocysts at E7, 8 and 9. Asterisks mark degenerating embryos. Scale bar, 200 µm. (F) Typical ICM outgrowth from bovine E9 blastocyst. Scale bar, 50 µm. (G) Summary of bovine AFX cell derivation from different blastocyst stages. (H) Teratoma from bovine AFX cells, stained with hematoxylin and eosin. Scale bar, 200 µm. (I) Immunostaining of sheep and cow AFX cells maintained with IWP2 instead of XAV939 for 8 passages. Scale bars, 100 µm.

Fig.S3

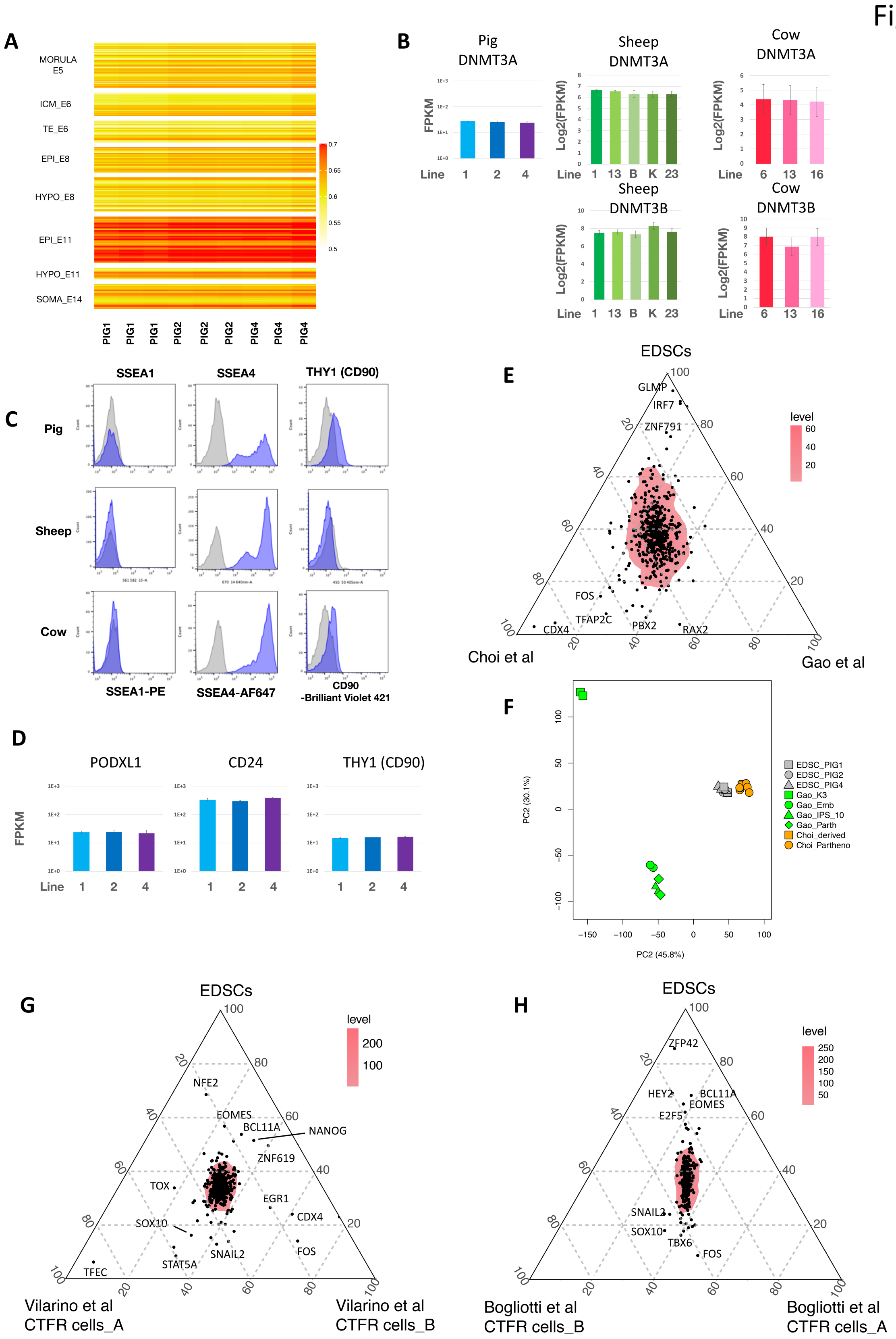

Development • Supplementary information

Fig. S3. Related to Fig. 3

(A) Heatmap showing Pearson correlation between porcine EDSCs and pig embryo stages for all expressed genes. (B) FPKM values for *DNMT3A* and *DNMT3B* in EDSCs from each species. *DNMT3B* expression in the pig embryo and EDSCs is shown in Fig. 3C. Error bars represent S.D. from triplicates. (C) Flow cytometry analysis of surface marker expression detected with conjugated antibodies. Dark grey peaks are control profiles without antibody and blue peaks are with antibody. (D) Log_2FPKM values from porcine EDSC RNA-seq for surface protein genes. (E) Ternary plot computed with E11 expressed transcription factor genes (Table S1). (F) PCA using all genes for porcine EDSCs and lines of Choi et al. (2019) and Gao et al. (2019) (G) Ternary plot analysis of sheep EDSCs and two lines of sheep CTFR cells (Vilarino et al., 2020) using highly expressed orthologous transcription factor genes from porcine E11 epiblast. (H) Ternary plot analysis as above for bovine EDSCs and CTFR lines (Bogliotti et al., 2018).

Fig.S4

A

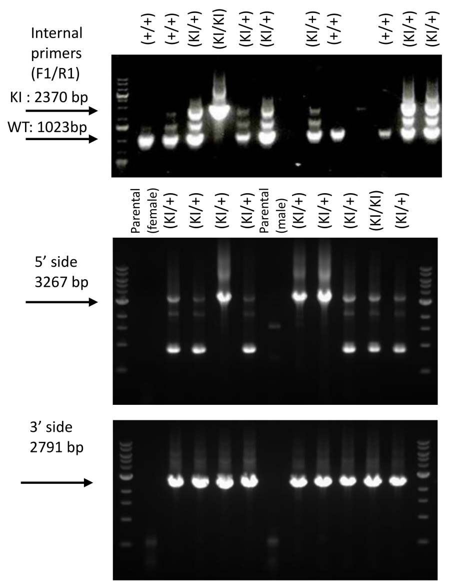

B

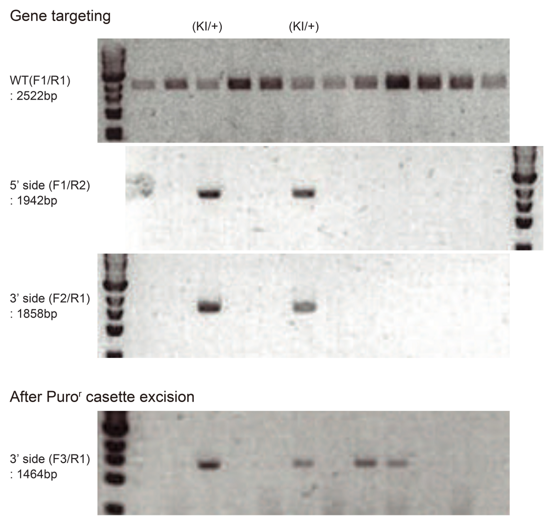

C

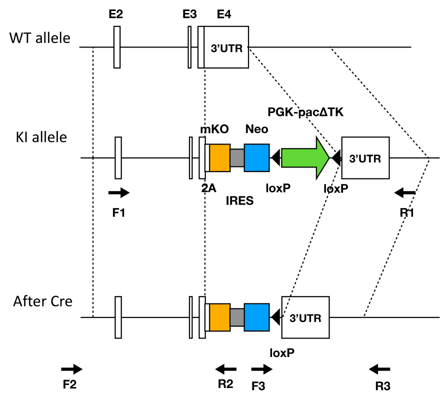

D

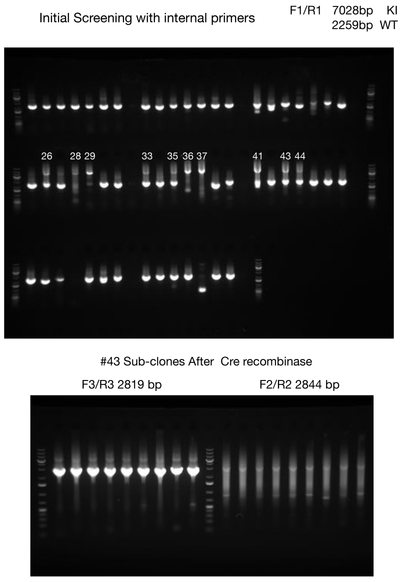

E

	Percentage of diploid cells (n=54)
Clone41-1	27/30 (90%)
Clone43-2	20/27 (74%)

F

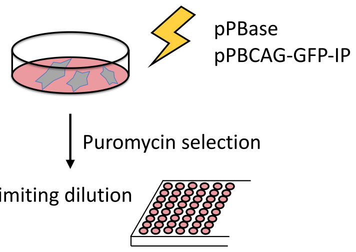

	Percentage of diploid cells (n=60)
Clone 1	9/13 (69%)
Clone 2	6/19 (33%)

G

pK1N3 (XY)
Passage: 15

pK2N3 (XX)
Passage: 22

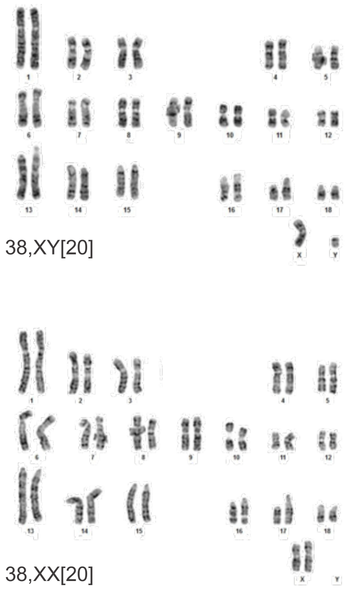

H

Donor cell: Name	Sex	Passage	Genotype	Enucleated oocyte	Electrofused oocyte (%)	Cleaved embryo (%)	Blastocyst (%)
pK1N3	XY	19	NANOS3 tdTomato	141	141(100)	80(58)	41(29)
pK2N3	XX	29	NANOS3 tdTomato	138	138(100)	69(50)	21(15)

Donor cell: Name	Sex	Passage	Genotype	Enucleated oocyte	Electrofused oocyte (%)	Cleaved embryo (%)	Blastocyst (%)	Fetus (%)
pK1N3	XY	20	NANOS3 tdTomato	214	210(98.1)	138(66)	58(27.8)	15(29)*

*5 fetuses are entirely normal

Fig. S4. Related to Fig.4

(A) Genomic PCR screening of *NANOG* targeting. Clones were screened for integration using internal primers and targeting was confirmed with primers external to both homology arms. (B) Genomic PCR screening of *NANOS3* targeting. (C) Sheep DPPA3 targeting strategy (D) Genotyping results. Initial screening was performed with primers F1 and R1 (upper gel). After clonal expansion, Cre recombinase was transiently transfected and sub-clones were expanded. The genotyping result for clone #43 sub-clones is presented (bottom gel). (E) Summary of chromosome counts for two sub-clones from two independent targeted clones (#41 and #43). (F) Stable transection of GFP reporter into bovine EDSCs and clonal expansion. After 7-10 days, 11 GFP colonies showing stem cell morphology were further expanded. Two clones were characterised by metaphase analysis. (G) G-banding analysis of NANOS3 targeted clones. (H) Summary of cloning experiments.

Table S1. List of transcription factor genes used for ternary plots

Click here to download Table S1

Table S2. List of primers

Click here to download Table S2

Table S3. List of antibodies

Click here to download Table S3
