# Supplementary material for: Pluripotent stem cells related to embryonic disc exhibit common self-renewal requirements in diverse livestock species
Source: Development. 2021 Dec 7;148(23):dev199901. doi: 10.1242/dev.199901 (PMC8714072; doi:10.1242/dev.199901)
Supplement: Reviewer comments [file develop-148-199901_review_history.pdf]

# Pluripotent stem cells related to embryonic disc exhibit common self-renewal requirements in diverse livestock species

Masaki Kinoshita, Toshihiro Kobayashi, Benjamin Planells, Doris Klisch, Daniel Spindlow, Hideki Masaki, Susanne Bornelöv, Giuliano Giuseppe Stirparo, Hitomi Matsunari, Ayuko Uchikaro, Ismael Lamas-Toranzo, Jennifer Nichols, Hiromitsu Nakauchi, Hiroshi Nagashima, Ramiro Alberio and Austin Smith
DOI: 10.1242/dev.199901

**Editor**: Patrick Tam

**Review timeline**

| | |
|---|---|
| Original submission: | 15 June 2021 |
| Editorial decision: | 19 July 2021 |
| First revision received: | 18 October 2021 |
| Accepted: | 26 October 2021 |

---

**Original submission**

First decision letter

MS ID#: DEVELOP/2021/199901

MS TITLE: Pluripotent stem cells corresponding to embryonic disc exhibit common self-renewal requirements in diverse livestock species

AUTHORS: Masaki Kinoshita, Toshihiro Kobayashi, Benjamin Planells, Doris Klisch, Daniel Spindlow, Hideki Masaki, Susanne Bornelov, Giuliano Stirparo, Hitomi Matsunari, Ayuko Uchikaro, Ismael Lamas-Toranzo, Jennifer Nichols, Hiromitsu Nakauchi, Hiroshi Nagashima, Ramiro Alberio, and Austin Smith

I have now received all the referees' reports on the above manuscript, and have reached a decision. The referees' comments are appended below, or you can access them online: please go to BenchPress and click on the 'Manuscripts with Decisions' queue in the Author Area.

As you will see, the referees express interest in your work, but have some criticisms and recommend a revision of your manuscript before we can consider publication. If you are able to revise the manuscript along the lines suggested (please also see Editor's note), which may involve further experiments, I will be happy to receive a revised version of the manuscript. Your revised paper will be re-reviewed by one or more of the original referees, and acceptance of your manuscript will depend on your addressing satisfactorily the reviewers' major concerns. Please also note that Development will normally permit only one round of revision.

We are aware that you may be experiencing disruption to the normal running of your lab that make experimental revisions challenging. If it would be helpful, we encourage you to contact us to discuss your revision in greater detail. Please send us a point-by-point response indicating where you are able to address concerns raised (either experimentally or by changes to the text) and where you will not be able to do so within the normal timeframe of a revision. We will then provide further guidance. Please also note that we are happy to extend revision timeframes as necessary.

Please attend to all of the reviewers' comments and ensure that you clearly highlight all changes made in the revised manuscript. Please avoid using 'Tracked changes' in Word files as these are lost

© 2021. Published by The Company of Biologists under the terms of the Creative Commons Attribution License (https://creativecommons.org/licenses/by/4.0/).

in PDF conversion. I should be grateful if you would also provide a point-by-point response detailing how you have dealt with the points raised by the reviewers in the 'Response to Reviewers' box. If you do not agree with any of their criticisms or suggestions please explain clearly why this is so.

Reviewer 1

*Advance summary and potential significance to field*

The manuscript entitled "Pluriopotent stem cells corresponding to embryonic disc exhibit common self-renewal requirements in diverse livestock species" by Kinoshita et al. analyses pluripotent embryonic disc-like stem cell lines from porcine, ovine, and bovine embryos and documents similarity among these and to embryonic disc stage embryos. For this the authors adopt previously established culture conditions for formative pluripotent stem cells based on FGF and Activin and beta-catenin inhibition by tankyrase inhibitors to establish cell lines from micro-dissected epiblasts. The authors go on to demonstrate the usability of their new pig EDSCs by showing stable transfection, clonal expansion, and the targeting of fluorescent reporters to the Nanog and Nanos loci. Finally, the reporter cells are used to obtain cloned pig embryos through nuclear transplantation. For all three species transcriptome profiling and in vitro differentiation is analysed. Although pluripotent stem cells from these species have been established previously the present study advances by comparing cell lines from different species in the same culture conditions. This allows the authors to describe similarities between the cell lines and to embryonic discs stage embryos.

The results suggest that embryonic disc like stem cells (EDSCs) represent a convergent pluripotent state of mammals that is amenable to culture. In addition, a close relation ship between EDSCs and formative pluripotent stem cells of rodents and primates is suggested. The discovery of EDSCs is conceptually important for the developmental biology of mammals. One limitation appears that there are few markers that would distinguish different states of pluripotency. The authors perform a detailed characterization based on transcriptome profiling and the presence of an inactive X chromosome. However, it appears a number of pluripotency associated genes are expressed and it remains unclear which genes are functionally relevant to determine embryonic disc stage cells. The new stem cell lines will be of high interest to further address this question in future. In addition applications in biotechnology and developmental biology will be facilitated through their differentiation potential in vitro. The experiments are performed to high technical standards and that data are convincing.

*Comments for the author*

A few suggestions might be considered for further improving the study.

Specific points

1. A major advance of this study is the dedection of an embryonic disc like stage of pluripotency in different species under comparable culture conditions. This is inferred from differentiation potential, X inactivation cell morphology, and expression profiling. However, this does not lead to a ready marker for clearly identifying the EDSCs. What makes these cells different from other stages of pluripotency that show overlap of associated transcription factors? Is lower Nanog expression of functional significance to make the cells EDSCs as opposed to primed or extended pluripotent SCs? Making EDSC state clearly identifiable among other states of pluripotency would strengthen the idea of EDSCs as a new pluripotent attractor state that is shared among different mammals.

2. The authors perform a series of experiments that demonstrate the ability to genetically modify and clonally expand porcine EDSCs. For the more distant bovine/ovine EDSCs in vitro differentiation is shown. Could a comment be added if the bovine/ovine cells can also clonally expand and amenable to genetic modification. These cells might be interesting for biotechnological applications.

3. The advance in the culture system could be positioned more specifically.
The present version leads to the impression that an existing culture system AFX with laminin/fibronectin has been applied in the study and the main advance could be made by using

© 2021. Published by The Company of Biologists under the terms of the Creative Commons Attribution License (https://creativecommons.org/licenses/by/4.0/).

micro-dissection and selection of the embryonic tissues. In particular page 8 relates EDSCs to formative pluripotent stem cells from rodents. From this it appear that similar culture conditions give cells line with distinct characteristics in different species, which is an important observation and the point could be made more clearly.

4. The discussion raises the important point of the ability of newly derived EDSCs for chimera formation. Could the authors add a brief mention if chimeras of farm animals have been obtained before and what the developmental biology could allow to demonstrate in future. Recently it has been shown that overcoming the chimera restriction in primate embryos required an earlier stage of pluripotency.

Minor points
a) The nomenclature variably referring to AFX cells and EDSCs at different sections makes it more difficult to follow their experiments. A clean separation of AFX culture conditions and prefixing the species of the EDSCs should be considered, especially in the figure legends. Also the cell line and the sex of the cell lines should be identified in the figure legends.
b) Figure 1F should show H3K27me3 staining of the inactive X. A magnified view is needed to make this visible. The panel in 1E seems to lack some values (EF) for TBXT and FCKA2 and should be checked. As initiation of X inactivation appears to be a independent marker for the developmental state could a Xi staining also be included for the ovine / bovine cells?
c) It would be interesting to hear the authors opinion on derivation of EDSCs by reprogramming instead from embryos. Could EDSCs be derived by iPSC reprogramming in AFX conditions?

<u>Reviewer 2</u>

*Advance summary and potential significance to field*

The manuscript by Kinoshita et al. describes the molecular and functional characterization of pluripotent stem cell lines derived from pig, sheep and bovine preimplantation embryos. This is a notoriously difficult task and this study appears to have taken an important step by describing pig cell lines capable of stably self-renewing for at least 10 passages in a defined culture medium. Ovine and bovine cell lines seem less stable and their characterization is more superficial.

*Comments for the author*

Major points
- Fig. 1B: I cannot see if the karyotype of the pig AFX line is indeed normal based on the picture provided. G-
banding analysis should be provided as in Figure S4C, especially since the karyotypes of the Nanos3 targeted clones seem to exhibit some chromosomal abnormalities (see comment below).
- Fig. 1C: Pictures of Oct4, Sox2 and Nanog immunofluorescence for AFX cells treated with MEK inhibitor are missing.
- Fig. 1F: Immunostaining of H3K27me3 foci in female cells is not clearly visible. A higher magnification picture should be provided.
- Fig. 2: It is not clear how many passages ovine and bovine AFX cells can withstand. Can they be passed beyond passage 10 like the porcine AFX cell lines?
- Fig. S4C: The karyotypes of the Nanos3 targeted clones do not seem normal. Both show abnormalities: in the pK1N3 line, the 2 chromosomes 13 and the 2 chromosomes 17 are not identical; In the pK2N3 line, the 2 chromosomes 17 also have different aspects. This questions the euploïdy the parental cells.
- Fig. 4 and S4D: Gestation rate after transfer of NT embryos (30%) is lower than that obtained with somatic cells (70%) in a previous study (Mao et al., Cell Reprogram, 2012). Have births been obtained? Can the chromosomal abnormalities that are visible on the S4C picture explain this drop in efficiency compared to previous works, including NT with pig iPS cells?

Minor points:
Page 5, end of 1st section: pSXX ??
The following references are not complete: Bogliotti et al., Choi et al., Hamilton & Ferry, Kinoshita et al., Mulas et al.,

© 2021. Published by The Company of Biologists under the terms of the Creative Commons Attribution License (https://creativecommons.org/licenses/by/4.0/).

Reviewer 3

*Advance summary and potential significance to field*

In this study, Kinoshita et al. describe a culture system that supports efficient derivation of pluripotent cell lines from porcine, ovine and bovine embryos. The authors demonstrate that the cell lines, which are derived from embryonic disc (or pre-implantation embryos that presumably progress to this stage in vitro), express genes characteristic of post-implantation but not pre-implantation epiblast. The cell lines appear to remain euploid during propagation in vitro (but see points 1 and 2 below), can undergo differentiation in vitro into progenitors representative of all three embryonic germ layers, form well differentiated teratomas, and in the case of the porcine cells, serve as donors for nuclear transfer experiments in which cloned embryos showed germline development.

Whilst this report is primarily technical in nature, it will be of interest to readers of this journal, who may wish to use the cell lines in comparative studies of mammalian development (it would be interesting to look at gastruloid formation, or extraembryonic or germline development in vitro; if the authors have done so already the data would strengthen the appeal and impact of the study), and stem cell and developmental biologists will be interested in the concept of a common attractor state corresponding to the embryonic disc stage in these species.

*Comments for the author*

As the authors note, elements of this culture system have been reported previously to support establishment of pluripotent stem cells from livestock species. This study reports a simple, defined system that reliably enables derivation of stem cell lines across three important domestic species. The evidence to support the claim of pluripotency is strong (chimera formation is not expected from these cells, which resemble post-implantation epiblast, but the teratoma studies are very convincing) and the system appears robust. The characterization of the cell lines is thorough, though more information on the exact number of lines examined would be useful (see below). Information on gastruloid formation or extraembryonic or germline differentiation in vitro would increase the impact of the study in the developmental biology community.

Specific questions:
1. L125-how many independent cell lines went through the characterization depicted in Figure 1 b,c d and g
2. L127-did cell lines remain euploid during long term cultivation?
3. L145-it would be helpful to examine expression of cell surface markers for post-implantation epiblast cells, e.g.Podocalyxin, CD24, TRA-1-60, others.
4. L206-does this refer to Supplemental Figure 2f?
5. L209-again how many independent cell lines from each species were assessed?
6. L216 same question, an important one since human cell lines can vary in their response to differentiation protocols.

---

**First revision**

Author response to reviewers' comments

**Point by point Response to Reviewers**

Reviewer 1 Advance Summary and Potential Significance to Field:
The manuscript entitled "Pluriopotent stem cells corresponding to embryonic disc exhibit common self-renewal requirements in diverse livestock species" by Kinoshita et al. analyses pluripotent embryonic disc-like stem cell lines from porcine, ovine, and bovine embryos and documents similarity among these and to embryonic disc stage embryos. For this the authors adopt previously established culture conditions for formative pluripotent stem cells based on FGF and Activin and beta-catenin inhibition by tankyrase inhibitors to establish cell lines from micro-dissected epiblasts.

© 2021. Published by The Company of Biologists under the terms of the Creative Commons Attribution License (https://creativecommons.org/licenses/by/4.0/). 

***The formative stem cell culture condition for mouse and human is different from AFX medium used here. FS cell medium does not contain FGF and includes RAR inhibition (Kinoshita et al., Cell Stem Cell, 2021)***

The authors go on to demonstrate the usability of their new pig EDSCs by showing stable transfection, clonal expansion, and the targeting of fluorescent reporters to the Nanog and Nanos loci. Finally, the reporter cells are used to obtain cloned pig embryos through nuclear transplantation. For all three species transcriptome profiling and in vitro differentiation is analysed. Although pluripotent stem cells from these species have been established previously, the present study advances by comparing cell lines from different species in the same culture conditions. This allows the authors to describe similarities between the cell lines and to embryonic discs stage embryos. The results suggest that embryonic disc like stem cells (EDSCs) represent a convergent pluripotent state of mammals that is amenable to culture. In addition, a close relation ship between EDSCs and formative pluripotent stem cells of rodents and primates is suggested. The discovery of EDSCs is conceptually important for the developmental biology of mammals. One limitation appears that there are few markers that would distinguish different states of pluripotency. The authors perform a detailed characterization based on transcriptome profiling and the presence of an inactive X chromosome. However, it appears a number of pluripotency associated genes are expressed and it remains unclear which genes are functionally relevant to determine embryonic disc stage cells. The new stem cell lines will be of high interest to further address this question in future. In addition applications in biotechnology and developmental biology will be facilitated through their differentiation potential in vitro. The experiments are performed to high technical standards and that data are convincing.

Reviewer 1 Comments for the Author:
A few suggestions might be considered for further improving the study. Specific points
1. A major advance of this study is the dedection of an embryonic disc like stage of pluripotency in different species under comparable culture conditions. This is inferred from differentiation potential, X inactivation, cell morphology, and expression profiling. However, this does not lead to a ready marker for clearly identifying the EDSCs. What makes these cells different from other stages of pluripotency that show overlap of associated transcription factors? Is lower Nanog expression of functional significance to make the cells EDSCs as opposed to primed or extended pluripotent SCs?

Making EDSC state clearly identifiable among other states of pluripotency would strengthen the idea of EDSCs as a new pluripotent attractor state that is shared among different mammals.

***Our data show that EDSCs lack early epiblast transcription factors that characterise mouse ES cells and human naïve PSCs. We also show lack of expression of early lineage markers as found in mouse EpiSCs. Our main finding therefore is that cells from the three species appear very similar and related to the intermediate stage of pluripotency in vivo, after naïve transition and prior to gastrulation onset. We agree that there are currently few markers to discriminate pluripotency progression along the formative trajectory, particularly in these livestock species. We refer to this limitation in the final section of the discussion: "In future studies it will be informative to examine the relatedness of EDSCs to FS cells, in terms of transcriptome features, chromatin organization, and functional attributes of chimaera colonization and germ cell formation. Positioning of EDSCs on the formative to primed pluripotency trajectory will also benefit from greater temporal resolution of mid to late epiblast transcriptome progression in embryos of the different species."***

2. The authors perform a series of experiments that demonstrate the ability to genetically modify and clonally expand porcine EDSCs. For the more distant bovine/ovine EDSCs in vitro differentiation is shown. Could a comment be added if the bovine/ovine cells can also clonally expand and amenable to genetic modification. These cells might be interesting for biotechnological applications.

***We now provide data on gene targeting in ovine EDSCs and stable transfection in bovine EDSCs, in both cases with clonal expansion (Figure S4C-F).***

3. The advance in the culture system could be positioned more specifically. The present version leads to the impression that an existing culture system AFX with laminin/fibronectin has been

© 2021. Published by The Company of Biologists under the terms of the Creative Commons Attribution License (https://creativecommons.org/licenses/by/4.0/).

applied in the study and the main advance could be made by using micro-dissection and selection of the embryonic tissues. In particular page 8 relates EDSCs to formative pluripotent stem cells from rodents. From this it appear that similar culture conditions give cells line with distinct characteristics in different species, which is an important observation and the point could be made more clearly.

***As noted above, AFX has not previously been applied to livestock PSC derivation. We have clarified the text referring to previous and recent reports that have used culture conditions with some overlapping components. Further investigations will be required to make reliable conclusions on the relationship of EDSCs to mouse EpiSCs and human PSCs cultured in similar medium. This is non-trivial due to genome divergence and the still incomplete genome assemblies for these animals.***

4. The discussion raises the important point of the ability of newly derived EDSCs for chimera formation. Could the authors add a brief mention if chimeras of farm animals have been obtained before and what the developmental biology could allow to demonstrate in future. Recently it has been shown that overcoming the chimera restriction in primate embryos required an earlier stage of pluripotency.

***There have been several reports over the years of chimaeras in farm animals, but none have proven reproducible or stand up to rigorous examination of the data. We would prefer not to divert into this contentious area which is tangential to the data we present because AFX cells are not naïve and therefore not predicted to have high chimaera competency.***

Minor points

a) The nomenclature variably referring to AFX cells and EDSCs at different sections makes it more difficult to follow their experiments. A clean separation of AFX culture conditions and prefixing the species of the EDSCs should be considered, especially in the figure legends. Also the cell line and the sex of the cell lines should be identified in the figure legends.

***We use the designation AFX cells prior to the demonstration of transcriptome identity with embryonic disc which we consider necessary to assign as EDSCs. We have adjusted figure 2 legends to be consistent. Following the reviewer suggestion, we now use species prefixes in the figure legends. We have not observed any consistent differences between cell lines or according to gender and therefore do not think it is necessary to complicate the legends with individual identities.***

b) Figure 1F should show H3K27me3 staining of the inactive X. A magnified view is needed to make this visible. The panel in 1E seems to lack some values (EF) for TBXT and FCKA2 and should be checked. As initiation of X inactivation appears to be a independent marker for the developmental state could a Xi staining also be included for the ovine / bovine cells?

***We have replaced Fig.1F with a higher magnification image.***
***In Fig. 1E, these two genes are not detected in fibroblast (EF) samples. We added N.D. to the figure and legend.***
***We have added H3K27me3 staining of female sheep EDSCs in Fig. S2. To date the bovine lines isolated are all male.***

c) It would be interesting to hear the authors opinion on derivation of EDSCs by reprogramming instead from embryos. Could EDSCs be derived by iPSC reprogramming in AFX conditions?

***We anticipate this will be the case and can be investigated in future studies. We have added a comment to the discussion "It may be anticipated that EDSCs can be derived by somatic cell reprogramming which would enable their generation from eilte livestock specimens."***

Reviewer 2 Advance Summary and Potential Significance to Field:
The manuscript by Kinoshita et al. describes the molecular and functional characterization of pluripotent stem cell lines derived from pig, sheep and bovine preimplantation embryos. This is a notoriously difficult task and this study appears to have taken an important step by describing pig

© 2021. Published by The Company of Biologists under the terms of the Creative Commons Attribution License (https://creativecommons.org/licenses/by/4.0/).

cell lines capable of stably self-renewing for at least 10 passages in a defined culture medium. Ovine and bovine cell lines seem less stable and their characterization is more superficial.

***We take the opportunity to clarify that, once established, EDSCs from the three species are similarly stable and we are able to passage them more than 30 times. Most of the experiments were performed between passage 10 to 25. We include additional characterisation of ovine and bovine lines in the revision.***

Reviewer 2 Comments for the Author: Major points

- Fig. 1B: I cannot see if the karyotype of the pig AFX line is indeed normal based on the picture provided. G-banding analysis should be provided as in Figure S4C, especially since the karyotypes of the Nanos3 targeted clones seem to exhibit some chromosomal abnormalities (see comment below).

***We performed G-banding analysis of passage 21 cells and replaced the figure 1B.***

- Fig. 1C: Pictures of Oct4, Sox2 and Nanog immunofluorescence for AFX cells treated with MEK inhibitor are missing.

***We did not show these data because MEK inhibition causes rapid collapse of the cultures. However, we repeated this experiment with two more lines and performed staining before complete loss of cells in MEK inhibitor (replacement Fig 1C). The original results for IWP-2 are now moved to Fig. S1D.***

- Fig. 1F: Immunostaining of H3K27me3 foci in female cells is not clearly visible. A higher magnification picture should be provided.

***We have replaced the image.***

- Fig. 2: It is not clear how many passages ovine and bovine AFX cells can withstand. Can they be passed beyond passage 10 like the porcine AFX cell lines?

***Yes, lines from all three species can be passaged more than 30 times. We added this point.***

- Fig. S4C: The karyotypes of the Nanos3 targeted clones do not seem normal. Both show abnormalities: in the pK1N3 line, the 2 chromosomes 13 and the 2 chromosomes 17 are not identical; In the pK2N3 line, the 2 chromosomes 17 also have different aspects. This questions the euploïdy the parental cells.

***We agree the G-banded chromosomes in this image may not be identical. However, pig chromosomes 13 and 17 are acrocentric chromosomes. Acrocentric chromosomes are known to have variation in the length of short arms between healthy individuals in human (Liehr, T., 2014, CG-CNVs. In Benign & Pathological Chromosomal Imbalances, pp. 13-24.), and may also vary in pigs.***

**[some comments/responses relating to unpublished findings have been removed]**

- Fig. 4 and S4D: Gestation rate after transfer of NT embryos (30%) is lower than that obtained with somatic cells (70%) in a previous study (Mao et al., Cell Reprogram, 2012). Have births been obtained? Can the chromosomal abnormalities that are visible on the S4C picture explain this drop in efficiency compared to previous works, including NT with pig iPS cells?

***Please see response to preceding question. Of note, current nuclear transfer protocols in pig are optimised for fibroblasts and include a cell cycle synchronisation step that cannot readily be applied to EDSCs. Therefore, further studies will be required to establish the best conditions and optimal efficiency for EDSC nuclear transfer. We now indicate this in the discussion.***

© 2021. Published by The Company of Biologists under the terms of the Creative Commons Attribution License (https://creativecommons.org/licenses/by/4.0/).

***We are not aware of published reports of successful cloning from porcine iPS cells. We have added citation to a large study (Fan et al., Cell Research, 2013) that reported no successful development from iPSCs. Those authors do report a single live neonate cloned from iPSCs after induction of differentiation. They ascribed these results to persisting transgene expression in undifferentiated porcine iPSCs, which may be silenced on differentiation.***
***Recent papers that have claimed cloning from bovine embryo-derived PSCs (Bogliotti et al.) or porcine expanded potential stem cells (Zhao et al.) only assessed blastocyst formation in vitro.***

Minor points:

Page 5, end of 1st section: pSXX ??
The following references are not complete: Bogliotti et al., Choi et al., Hamilton & Ferry, Kinoshita et al., Mulas et al.,

***Thank you for pointing out these errors which are now corrected.***

Reviewer 3 Advance Summary and Potential Significance to Field:
In this study, Kinoshita et al. describe a culture system that supports efficient derivation of pluripotent cell lines from porcine, ovine and bovine embryos. The authors demonstrate that the cell lines, which are derived from embryonic disc (or pre-implantation embryos that presumably progress to this stage in vitro), express genes characteristic of post-implantation but not pre-implantation epiblast. The cell lines appear to remain euploid during propagation in vitro (but see points 1 and 2 below), can undergo differentiation in vitro into progenitors representative of all three embryonic germ layers, form well differentiated teratomas, and in the case of the porcine cells, serve as donors for nuclear transfer experiments in which cloned embryos showed germline development.

Whilst this report is primarily technical in nature, it will be of interest to readers of this journal, who may wish to use the cell lines in comparative studies of mammalian development (it would be interesting to look at gastruloid formation, or extraembryonic or germline development in vitro; if the authors have done so already the data would strengthen the appeal and impact of the study), and stem cell and developmental biologists will be interested in the concept of a common attractor state corresponding to the embryonic disc stage in these species.

Reviewer 3 Comments for the Author:
As the authors note, elements of this culture system have been reported previously to support establishment of pluripotent stem cells from livestock species. This study reports a simple, defined system that reliably enables derivation of stem cell lines across three important domestic species. The evidence to support the claim of pluripotency is strong (chimera formation is not expected from these cells, which resemble post-implantation epiblast, but the teratoma studies are very convincing) and the system appears robust. The characterization of the cell lines is thorough, though more information on the exact number of lines examined would be useful (see below). Information on gastruloid formation or extraembryonic or germline differentiation in vitro would increase the impact of the study in the developmental biology community.

***See point below for numbers of cell lines. We agree that it may be interesting to generate gastruloids from EDSCs. However, that will require some methods work up prior to analysis, which is beyond the scope of this paper.***

Specific questions:

1. L125-how many independent cell lines went through the characterization depicted in Figure 1 b,c d and g

***We examined three different lines (at p8, p8, p21 respectively) by metaphase analysis and in each case counted a modal number of 38 chromosomes. We submitted the p21 sample for G-banding analysis and now provide this image in Fig 1B. Data in Fig 1C were originally from one line. We have now repeated this experiment with two more lines. We replaced the image in***

© 2021. Published by The Company of Biologists under the terms of the Creative Commons Attribution License (https://creativecommons.org/licenses/by/4.0/).

***Fig1C to add missing immunofluorescent stainings and moved the IWP2 results to Fig. S1D. Immunostainings in Fig 1D have been reproduced in six porcine lines. We tested teratoma formation from one line only to reduce use of animals in line with NC3R principles.***

2. L127-did cell lines remain euploid during long term cultivation?

***Yes, see response above and also data on chromosome counts after genetic manipulation and clonal expansion (Fig S4).***

3. L145-it would be helpful to examine expression of cell surface markers for post- implantation epiblast cells, e.g.Podocalyxin, CD24, TRA-1-60, others.

***We carried out flow cytometry analysis for SSEA1, SSEA4, TRA1-60, TRA1-80, CD24, CD57 and CD90. However, we only obtained reliable signal for SSEA4. At least for some cases this is likely due to poor species cross-reactivity of antibodies. Notably, RNA-seq data show appreciable expression of CD24 mRNA in pig EDSCs. We have added data for SSEA4 staining in Fig. S3B and provided FPKM data for cell surface marker genes in Fig. S3C.***

4. L206-does this refer to Supplemental Figure 2f?

***Yes. We have corrected the figure call out.***

5. L209-again how many independent cell lines from each species were assessed?

***We examined at least three independent cell lines from each species for alkaline phosphatase and for co-expression of OCT4, SOX2 and NANOG (Fig. 2A).***

6. L216 same question, an important one since human cell lines can vary in their response to differentiation protocols.

***For neural and endoderm differentiation, we tested two pig lines, three sheep lines and 2 cow lines and observed similar responses. For paraxial mesoderm, we tried at least two lines in each species at least twice. The efficiency was variable between experiments but did not appear dependent on cell line.***

***We have added numbers of lines tested to the figure legend or method.***

---

Second decision letter

MS ID#: DEVELOP/2021/199901

MS TITLE: Pluripotent stem cells related to embryonic disc exhibit common self-renewal requirements in diverse livestock species

AUTHORS: Masaki Kinoshita, Toshihiro Kobayashi, Benjamin Planells, Doris Klisch, Daniel Spindlow, Hideki Masaki, Susanne Bornelov, Giuliano Stirparo, Hitomi Matsunari, Ayuko Uchikaro, Ismael Lamas-Toranzo, Jennifer Nichols, Hiromitsu Nakauchi, Hiroshi Nagashima, Ramiro Alberio, and Austin Smith
ARTICLE TYPE: Research Article

I am happy to tell you that your manuscript has been accepted for publication in Development, pending our standard ethics checks.

© 2021. Published by The Company of Biologists under the terms of the Creative Commons Attribution License (https://creativecommons.org/licenses/by/4.0/).

Reviewer 1

*Advance summary and potential significance to field*

The revised verion of the manuscript entitled "Pluripotent stem cells related to embryonic disc exhibit common self-renewal requirements in diverse livestock species" by Kinoshita et al. contains additional data and clarifications in the text that have further strengthened the study In particular, the authors now provide evidence for genetic modification and clonal propagation of some of their bovine and ovine stem cell lines. These data demonstrate the usefulness and suggest that indeed stem cells from the three different species have common properties. New images now show the inactive X and karyotypes of the cells in a much improved presentation. In addition, the authors have changed the text that now positions their study well and illustrates their advance over earlier literature. The revision has addressed all my earlier concerns in a comprehensive and satisfactory manner.

*Comments for the author*

Minor points
a) I was wondering if the authors would have tired to black JAK signaling to see if these cells are independent of LIF as would be expected.
b) The revised text places the EDSCs in between FS and EpiSCs in mice. As these are very recent additions to the pluripotent states, including a characteristic to distinguish eg the ability for germ line differentiation of FS not so much in EpiSC would allow the reader to understand the biology better. Provided that the AFX medium has not been used on rodent and human cells one cannot avoid to wonder if AFX would allow to culture a pluripotent state of mice and what this would correspond to. As the states seem to converge in Laurasiatheria, would EDSC - like cells also exist in mice?

Reviewer 2

*Advance summary and potential significance to field*

See first review

*Comments for the author*

The authors have adequately responded to all my comments

Reviewer 3

*Advance summary and potential significance to field*

See initial review.

*Comments for the author*

The authors have provided satisfactory responses to all reviewers' queries.

© 2021. Published by The Company of Biologists under the terms of the Creative Commons Attribution License (https://creativecommons.org/licenses/by/4.0/).
